# Mechanistic Wound Healing and Antioxidant Potential of *Moringa oleifera* Seeds Extract Supported by Metabolic Profiling, In Silico Network Design, Molecular Docking, and In Vivo Studies

**DOI:** 10.3390/antiox11091743

**Published:** 2022-09-01

**Authors:** Nourhan Hisham Shady, Nada M. Mostafa, Shaimaa Fayez, Islam M. Abdel-Rahman, Sherif A. Maher, Ahmed Zayed, Entesar Ali Saber, Manal M. Khowdiary, Mahmoud A. Elrehany, Mubarak A. Alzubaidi, Faisal H. Altemani, Ahmed M. Shawky, Usama Ramadan Abdelmohsen

**Affiliations:** 1 Department of Pharmacognosy, Faculty of Pharmacy, Deraya University, Universities Zone, New Minia City 61111, Egypt; 2 Department of Pharmacognosy, Faculty of Pharmacy, Ain Shams University, Cairo 11566, Egypt; 3 Department of Pharmaceutical Chemistry, Faculty of Pharmacy, Deraya University, Minia 61519, Egypt; 4 Department of Biochemistry, Faculty of Pharmacy, Deraya University, Universities Zone, New Minia City 61111, Egypt; 5 Pharmacognosy Department, College of Pharmacy, Tanta University, Elguish Street (Medical Campus), Tanta 31527, Egypt; 6 Institute of Bioprocess Engineering, Technical University of Kaiserslautern, Gottlieb-Daimler-Straβe 49, 67663 Kaiserslautern, Germany; 7 Department of Histology and Cell Biology, Faculty of Medicine, Minia University, Minia 61519, Egypt, Delegated to Deraya University, Universities Zone, New Minia City 61111, Egypt; 8 Chemistry Department, Faculty of Applied Science, Umm Al-Qura University, Al-Lith Branch, Makkah 24211, Saudi Arabia; 9 Department of Biochemistry, Faculty of Medicine, Minia University, Minia 61519, Egypt; 10 Department of Biological Sciences, Faculty of Science, King Abdulaziz University, Jeddah 21589, Saudi Arabia; 11 Department of Medical Laboratory Technology, Faculty of Applied Medical Sciences, University of Tabuk, Tabuk 71491, Saudi Arabia; 12 Science and Technology Unit (STU), Umm Al-Qura University, Makkah 21955, Saudi Arabia; 13 Department of Pharmacognosy, Faculty of Pharmacy, Minia University, Minia 61519, Egypt

**Keywords:** wound healing, *Moringa*, molecular docking, drug likeness, network screening, antioxidant activity

## Abstract

*Moringa oleifera* Lam. (*Moringaceae*) is an adaptable plant with promising phytoconstituents, interesting medicinal uses, and nutritional importance. Chemical profiling of *M. oleifera* seeds assisted by LC-HRMS (HPLC system coupled to a high resolution mass detector) led to the dereplication of 19 metabolites. Additionally, the wound healing potential of *M. oleifera* seed extract was investigated in male New Zealand Dutch strain albino rabbits and supported by histopathological examinations. Moreover, the molecular mechanisms were investigated via different in vitro investigations and through analyzing the relative gene and protein expression patterns. When compared to the untreated and MEBO^®^-treated groups, topical administration of *M. oleifera* extract on excision wounds resulted in a substantial increase in wound healing rate (*p* < 0.001), elevating *TGF-β1*, VEGF, Type I collagen relative expression, and reducing inflammatory markers such as *IL-1β* and *TNF-α*. In vitro antioxidant assays showed that the extract displayed strong scavenging effects to peroxides and superoxide free radicals. In silico studies using a molecular docking approach against TNF-*α*, TGFBR1, and IL-1*β* showed that some metabolites in *M. oleifera* seed extract can bind to the active sites of three wound-healing related proteins. Protein–protein interaction (PPI) and compound–protein interaction (CPI) networks were constructed as well. Quercetin, caffeic acid, and kaempferol showed the highest connectivity with the putative proteins. In silico drug likeness studies revealed that almost all compounds comply with both Lipinski’s and Veber’s rule. According to the previous findings, an in vitro study was carried out on the pure compounds, including quercetin, kaempferol, and caffeic acid (identified from *M. oleifera*) to validate the proposed approach and to verify their potential effectiveness. Their inhibitory potential was evaluated against the pro-inflammatory cytokine IL-6 and against the endopeptidase MMPs (matrix metalloproteinases) subtype I and II, with highest activity being observed for kaempferol. Hence, *M. oleifera* seeds could be a promising source of bioactive compounds with potential antioxidant and wound healing capabilities.

## 1. Introduction

Herbal medicines are continuing to be a potential source of novel and unique drugs required for human welfare [1,2]. They provide chemical scaffolds for the development of synthetic and/or semi-synthetic analogues needed for the management of multiple disorders [3]. The recent advances in metabolomics [4], molecular biology [5], phytochemical analysis, and drug discovery [6] encouraged the natural products chemists to investigate the old therapeutic hypotheses and explore the mechanisms of the traditionally prescribed herbal medicines including those recorded in Chinese herbal medicines (CHMs), Ayurveda, and Thai traditional medicine (TTM) [7,8,9]. *Moringa oleifera* Lam. tree of the monogeneric family *Moringaceae* is native to the tropical Northern India, from where it has been spread and cultivated in many parts of the World specially in the Mediterranean basin and the Red Sea area, owing to its ability to adapt to the different climatic conditions [10,11] as well as its outstanding medicinal uses and nutritional benefits [12]. Its leaves, flowers, seeds, pods, bark, and roots have long been used by the Indians and Africans, i.e., the leaves and roots as anti-coagulants for the treatment of snake bites due to their content of thrombin and plasmin like proteases. The seed oil has been used for skin and hair care due to its antiseptic, anti-inflammatory, antioxidant, and nutritive values owing to the presence of tannins, saponins, flavonoids, vitamins, minerals, terpenoids, and glycosides [13].

In contrast to the leaves, Moringa seeds are pharmacologically less investigated despite their richness with essential oil (up to 40%—commercially known as “Ben oil” or “Behen oil”) [14], proteins (especially sulfated amino acids), carbohydrates (27.5%), minerals (e.g., Mg and Ca), vitamins (A and E), fatty acids (e.g., oleic acid), phenolic compounds (e.g., quercetin and *p*-hydroxybenzoic acid), phytosterols (e.g., *β*-sitosterol, stigmasterol, and campesterol), alkaloids, tannins, and saponins [15]. 

Since wounds affect the integrity of the skin and mucous membranes potentially subjecting the body to microbes, heat, light, and injury [16], immunity is stimulated to start an exceptionally multifaceted repairing process, named “wound healing”. The later consists of four consecutive (and sometimes) overlapping phases, including exudative/ inflammatory, resorptive, proliferative, and regenerative/ remodeling phases, which involve more than 30 signaling molecules, among them cytokines, chemokines, growth factors, macrophages, platelets, fibroblasts, and leukocytes [17]. Nevertheless, these phases were found to be facilitated when using dressings impregnated with natural products having anti-inflammatory, antioxidant, pro-collagen synthesis enhancers, and antibacterial activities [16].

Several wound healing studies involving *M. oleifera* extracts have been reported. For example, the ethyl acetate fraction of their leaves promoted fibroblasts proliferation [18]. Other studies revealed that the aqueous fraction showed better enhancement of the proliferation, migration, and viability of the dermal fibroblasts owing to the bioactive metabolites like vicenin-2 [19]; also, the seeds polysaccharides and their silver-derived nanoparticles promoted the migration of fibroblasts as evidenced by RT-PCR and histological analysis [20].

Herein, the antioxidant and wound healing ability of *M. oleifera* seeds were assessed and supported by in vitro and in vivo studies as well as the histopathological consequences following the application of the seeds extract. Furthermore, metabolic profiling was performed to underline the bioactive compounds involved in the activity. An in silico molecular docking study was performed on all the identified components and screened against three different protein targets which are involved in the process of wound healing (TNFα, TGFBR1, and IL-1ß). An in silico evaluation of the compounds’ drug likeness as well as the construction of protein–protein interactions (PPI) and compound–protein interactions (CPI) networks were performed.

## 2. Material and Methods

### 2.1. Plant Material

The seeds of *Moringa oleifera* were collected in January 2021 from the Sohag area in Egypt. It was authenticated by Prof. Dr. Nasser Barakat, Faculty of Science, Minia University, Egypt. A voucher specimen (Mor-1-2021) was archived at the Pharmacognosy Department, Faculty of Pharmacy, Deraya University, Egypt.

### 2.2. Extraction of Moringa oleifera Seeds

One kilogram of dried *Moringa oleifera* seeds was extracted by maceration in methanol at room temperature three times until exhausted. The alcoholic extract was concentrated under vacuum to yield a viscous syrupy residue (100 g).

### 2.3. Metabolomic Analysis

LC-MS was carried out using a Synapt G2 HDMS quadrupole time-of-flight hybrid mass spectrometer (Waters, Milford, CT, USA). The sample (2 μL) was injected into the BEH C_18_ column, adjusted to 40 °C, and connected to a guard column. A gradient elution of mobile phase was used, starting from 100% water in 0.1% formic acid as solvent A to 100% acetonitrile in 0.1% formic acid as solvent B. MZmine 2.12 (San Diego, CA, USA) was employed for differential investigation of MS data, followed by converting the raw data into positive and negative files in mzML format with ProteoWizard (Palo Alto, CA, USA) 

### 2.4. Molecular Docking Study 

The X-ray crystallographic structures of the protein targets catalytic domains, in complex with their co-crystalized ligands, were retrieved from the Protein Data Bank (http://www.rcsb.org/pdb) (accessed on 10 April 2022). All molecular modelling calculations and docking studies were carried out using ‘Molecular Operating Environment 2019.0102 software (MOE)’ (Sherbrooke St. West, Montreal, QC, Canada).

The preparation of the protein was carried out through the removal of water molecules as well as the uninvolved ligands using the quick preparation tool in MOE. Docking of the target compounds was performed after enzyme preparation. The following methodology was applied: The enzyme active site was assigned using the ‘site finder’ tool. The program specifications were adjusted to ligand atoms as the docking site and the alpha triangle as the placement methodology to be used. The scoring methodology London dG was adjusted to its default values. The MDB file of the ligand was loaded, and the docking calculations were run automatically. Receptor–ligand interactions of the complexes were examined in 2D and 3D styles. Those poses that showed the best ligand–enzyme interactions were selected and stored for energy calculations. Poses selection was done according to their binding scores and their RMSD values.

### 2.5. Animal Model

Twenty-four adult male New Zealand Dutch strain albino rabbits were purchased from the Faculty of Pharmacy, Deraya University, Minia, Egypt having an age of six months and weighing from 1 to 1.2 kg. The rabbits were kept in separate cages and were given a standard diet and tap water under controlled settings of 25 °C and 55% humidity with 12 h cycle of dark and light. Extreme care was taken and all procedures on rabbits were adopted according to Deraya laboratory guidelines. The approval code is 6/2021.

Methodology for wound excision model, wound healing evaluation, histopathological examination, statistical analysis are described in the Supplementary File.

Moreover, Real-Time Polymerase Chain Reaction is discussed in detail in the Supplementary Materials, as mentioned in Table S1. 

### 2.6. In Vitro IL-6, MMP-1, and MMP-2 Determinations

The in vitro determination of IL-6 and matrix metalloproteinases (MMP-1) and (MMP-2) were carried out quantitatively using ELISA kits having catalog numbers of ab178013 Human IL-6, PicoKine™ ELISA MBS175893, ab100606-MMP2 (Houston, TX, USA) respectively, according to the procedures described by the manufacturer. Results were presented as pg/mL ± SD for triplicate measurements.

## 3. Results and Discussion

### 3.1. QTOF-MS Assisted Dereplication of the Chemical Constituents in M. oleifera Seed Extract

Metabolomic profiling assisted by HR-LC-MS analysis of the crude extract of *Moringa oleifera* seeds (Table 1), as well as in-depth survey of literature, led to the identification of a wide array of secondary metabolites belonging to different phytochemical classes. 

### 3.2. Wound Closure Process

#### Estimation of the Wound Closure Rate 

Healing process is a complicated process that involves the restoration of tissue structure in wounded tissue [31,32]. Dermal wound repair has three phases: an inflammatory phase caused by pro-inflammatory mediator secretion and immune system suppression; a proliferative phase caused by fibroblast proliferation, collagen growth, and the development of new blood vessels; and a remodeling phase that includes regeneration and wounded tissue repair [33,34]. As a result, medications that might speed up wound healing with a possible input in all stages of the process will be required for efficient therapy, specifically those with low costs and less side effects.

**Figure 1:**
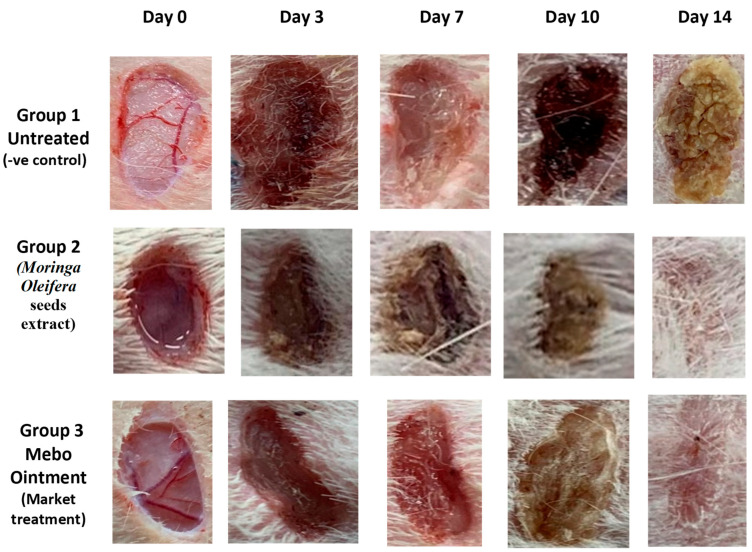
The wound healing potential of *Moringa oleifera* seeds extract compared to MEBO^®^ in excisional wounds on days 0, 3, 7, 10, and 14 post wounding. Rabbits were divided into three groups: group 1 was left untreated (-ve control), group 2 received *Moringa oleifera* seeds extract, and group 3 received MEBO^®^ (market treatment- + ve control).

Our findings revealed that wound closure in all experimental groups increased in a time-dependent manner. On the third post-injury day, the wound closure rate ranged from 7 to 16% in each group, with the untreated group having the lowest rate and the treated group having the highest one with no significant difference (*p* > 0.001) between the groups. Wound closure in the group treated with *M. oleifera* seeds extract reached 45% on the 7th day post treatment, which proved to be significantly higher (*p* < 0.001) than the untreated group (Figure 1).

**Figure 2:**
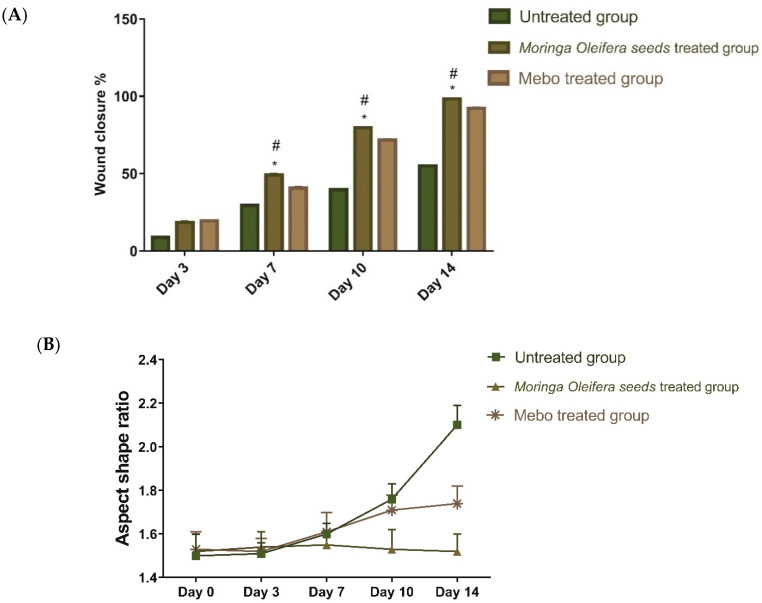
(**A**). Wound closure rates over time post-injury in all investigated groups. Group 1 was left untreated (-ve control). Group 2 received *Moringa oleifera* seeds extract. Group 3 received MEBO^®^ (marketed treatment- + ve control over 0, 3, 7, 10, and 14 days). After normalizing the variables using the Shapiro–Wilk test, a two-way ANOVA test was used to examine significant differences between groups. The expression of the data was expressed as mean ± SD. In comparison to the respective day’s untreated group, * *p* < 0.001; in comparison to the respective day’s MEBO^®^ group, # *p* < 0.001. (**B**) Wound aspect ratio was determined to describe observed changes in the shape and direction of wound contraction between groups (length:width).

On the 3rd-day post treatment, the group administered *M. oleifera* seeds extract showed quicker wound closure rates compared to that one treated with MEBO^®^ (38%) (*p* < 0.001). On the 10th day post treatment, the wound closure rate of the group treated with *M. oleifera* seeds extract (70%) was significantly higher (*p* < 0.001) than the group received no treatment (37%). On the 14th day post-punch, the wound in the group treated with *M. oleifera* seeds extract was entirely healed, and the incision was closed (96%) compared to 91% in the MEBO^®^-treated group (Figure 2). 

**Figure 3:**
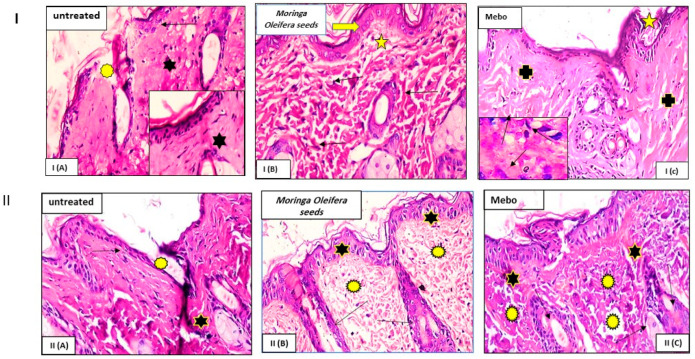
Wounded skin 7 (I-top) and 14 days (II-down) after incision. (**I**) Group I (**A**) (untreated): Showing a normal edged wound with normal epidermis (arrow). The wound is packed with blood clots (asterisk), which are highlighted by sloughed granulation tissue and dense uneven collagen bundles (star). Group I (**B**) (*Moringa oleifera* seeds extract) exhibited significant re-epithelization (thick arrow). The granulation tissue filling the defect’s base (from below) is mostly cellular (star). Collagen bundles manifested as disordered coarse and wavy bundles (arrows). Group I (**C**) (MEBO^®^): Scar tissue obstructing the wound (star). Collagen bundles form a reticular pattern around the defect, similar to that of the nearby normal dermis (crosses). The inset depicts inflammatory cellular infiltration, primarily of macrophages (black arrows). (**II**) Group II (**A**) (left untreated): Wide wound area (stars) with substantial inflammatory cellular infiltration in an acidophilic matrix (asterisks) and normal skin (arrows). Group II (**B**) (*Moringa oleifera* seeds extract): Typical stratified squamous keratinized epithelium (stars) and dermal matrix with coarse wavy collagen bundles pointing in various directions (asterisks). The freshly created hair follicles (arrows). Group II (**C**) (MEBO^®^): Typical epithelium, thin scar tissue extending into the dermis (stars), and reticular dermis with coarse wavy collagen bundles organized in different orientations are all visible (asterisks), and newly formed hair follicles (arrows). (Hx and E stain × 200 and 400).

Wound closure is characterized as the centripetal flow of the edges of a full-thickness wound to help in wound tissue closure [35,36,37]. This was likewise supported by the histopathological examination where the injured tissue treated with the extract showed marked re-epithelization, granulation tissue filling the wound and formation of collagen bundles (Figure 3). Therefore, wound healing is a signal of re-epithelialization, granulation, angiogenesis, fibroblast proliferation, differentiation, and proliferation of keratinocytes [37].

### 3.3. Effect of Moringa oleifera Seeds Extract on the Expression of TGF-β, TNF-α, and IL-1β

**Figure 4:**
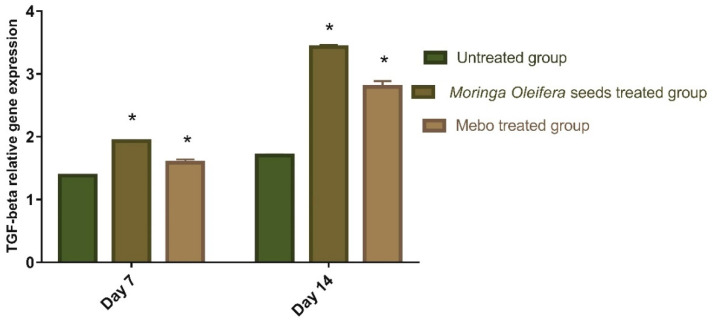
Gene expression in wound tissues for rabbits of different groups via quantitative RT-PCR. Data represent fold change relative to the normal control group expression after normalization to glyceraldehyde 3-phosphate dehydrogenase (GAPDH). Bars represent mean ± SD. Significant difference between groups is analyzed by a two-way ANOVA test, where: * *p* < 0.001 compared to those of the untreated group on the respective day.

Figure 4 depicted the mRNA expression of *TGF-β* following excisional wound therapy with *Moringa oleifera* seeds extract and MEBO^®^. TGF-*β* mRNA expression in skin tissues was substantially higher in wounds treated with *M. oleifera* seeds extract for 7 to 14 days compared to the untreated groups (*p* < 0.001). The relative gene expression of *M. oleifera* seeds extract-treated wounds showed a significant rise in the markers expression as compared to the MEBO^®^-treated group. The complicated connections between cells and the multiple growth factors are necessary for wound healing [38]. The most critical point of wound healing phases is when *TGF-β* recruits and activates inflammatory cells like neutrophils and macrophages during the hemostasis and inflammation phase. However, during the proliferative phase, it induces a variety of cellular responses, including re-epithelialization, angiogenesis, the development of granulation tissue, and the deposition of extracellular matrix [39,40]. During the remodeling phase, it promotes fibroblasts to grow and differentiate into myofibroblasts, which aid in wound closure [39,41,42]. Chronic non-healing wounds often result in a failure of *TGF-β* warning, whereas Feinberg and his colleagues [43] reported that *TGF-β* has an inhibitory impact on the production of collagenases that degrade collagen and extracellular matrix. These are coherent with the above measurements, which established that *M. oleifera* seeds extract enhanced *TGF-β* expression and hence recovered wound healing. The mRNA gene expression investigation of the injured tissues produced an increment in *TGF-β* levels in *M. oleifera* seeds extract-treated wound tissues relative to the untreated wound tissues. This may recommend that *M. oleifera* seeds extract upregulates the expression of *TGF-β* in the wound tissues.

**Figure 5:**
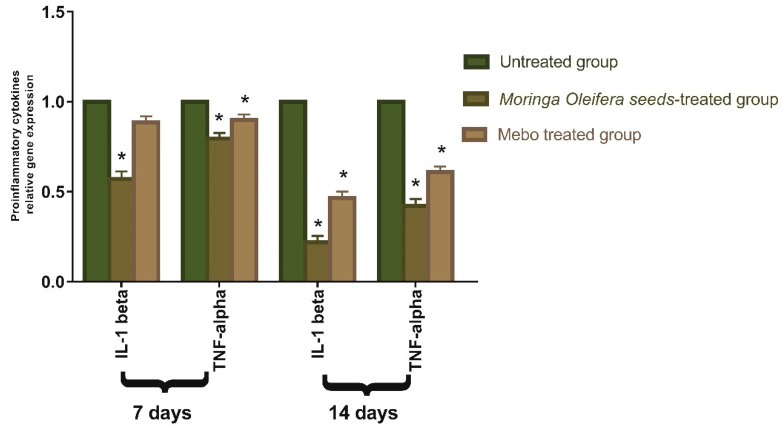
Gene expression in injured tissues of rabbits from various groups after normalizing to glyceraldehyde 3-phosphate dehydrogenase (GAPDH). The bars reflect the mean ± SD. A two-way ANOVA test is used to determine whether there is a significant difference between groups where: * *p* < 0.001 compared to those of the group left without treatment on that particular day.

As shown in Figure 5, the mRNA expression of *TNF-α* and *IL-1β* was illustrated. Analysis of mRNA expression of full-thickness wounded samples on day 7 post-injury revealed that the activity of the inflammatory markers *TNF-α* and *IL-1β* was significantly down-regulated in wounds treated with *M. oleifera* seeds extract or MEBO^®^ compared to the untreated wounds. However, wounded rabbits treated with *M. oleifera* seeds extract displayed more reduction in the inflammatory markers (*TNF-α*, and *IL-1β*) compared to the MEBO^®^-treated group. 

Moreover, *M. oleifera* seeds extract or MEBO^®^ treatment over 14 days showed a significantly decrease in *TNF-α* and *IL-1β* mRNA expression when compared to the untreated group at (*p* < 0.001). The expression of *TNF-α* and *IL-1β* in wounds treated with *M. oleifera* seeds extract were significantly lower than in the group treated with MEBO^®^. Pro-inflammatory cytokines (*IL-1β* and *TNF-α*) must be expressed appropriately to recruit neutrophils to the wound site. They are also identified as dynamic inducers of metalloproteinase (MMP) production in inflammatory and fibroblast cells. MMP destroys and eliminates damaged extracellular matrices (ECM) during wound healing to help in wound repair [44]. However, a prolonged inflammatory phase interferes with the healing process, and these cytokines and proteinase harm the tissue and contribute to the formation of chronic wounds [45]. 

*TNF-α* stimulates NF-κB, which in turn promotes gene expression of a plethora of pro-inflammatory cytokines and proteases such as MMP [46]. Therefore, suppressing inflammatory cytokines (*TNF-α* and *IL-1β*) by *M. oleifera* seeds extract can inhibit continued inflammation and enhance wound repair. The results of in vivo studies, which were demonstrated by a significant alteration in the mRNA expression of TGF-*β* and the inflammatory markers (TNF-α and IL-1*β*), are confirmed by the binding modes and free energies of the isolated compounds obtained during molecular docking studies within active sites of TGFBR1, TNF-α, and IL-1β). These results suggested that *M. oleifera* seeds extract could accelerate the switching process from inflammatory to anti-inflammatory responses.

### 3.4. In Vitro Antioxidant Assessment of Moringa oleifera Seeds Extract 

#### 3.4.1. Hydrogen Peroxide Scavenging Activity

**Figure 6:**
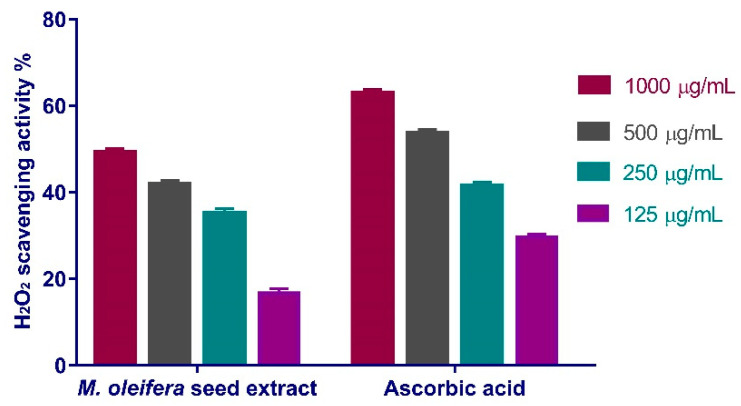
H_2_O_2_ radical scavenging activity of *M. oleifera* seeds extract at different concentrations (1000 µg/mL, 500 µg/mL, 250 µg/mL, and 125 µg/mL). Bars represent mean ± SD. Significant difference between groups is analyzed by a Two-way ANOVA test.

The maximal peroxide scavenging effect of *M. oleifera* seeds extract was 49.26% at concentration of 1000 µg/mL. When compared to the standard ascorbic acid (IC_50_ = 167.3 μg/mL), the extract reduced the generation of peroxide radicals in a dose-dependent manner (IC_50_ of 163.2 μg/mL) (Figure 6).

#### 3.4.2. Superoxide Radical Scavenging Activity 

**Figure 7:**
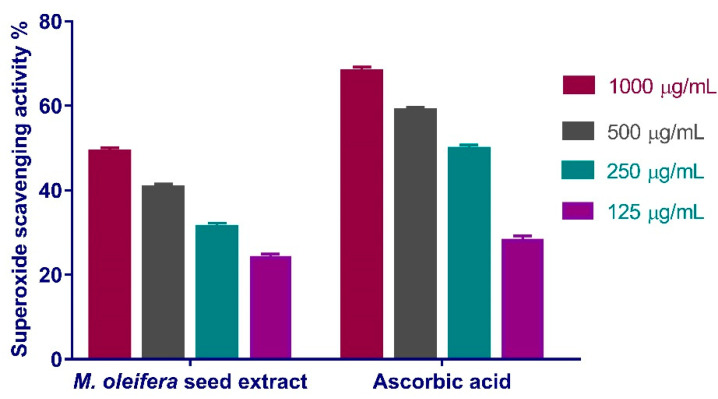
Superoxide radical scavenging activity of *Moringa oleifera* seeds extract at different concentrations (1000, 500, 250, and 125 µg/mL). Bars represent mean ± SD (standard deviation). Significant difference between groups is analyzed by a Two-way ANOVA test.

Similarly, the scavenging activity of the standard and the extract rise steadily with concentration (Figure 7). The maximum effect was observed at extract concentration of 1000 μg/mL where a 49.06% superoxide scavenging effect was observed. The concentration of *M. oleifera* seeds extract needed for 50% inhibition (IC_50_) was ca. 144.1 µg/mL, whereas that for the standard ascorbic acid was 156.7 μg/mL.

### 3.5. Molecular Docking Study 

In silico molecular docking study was carried out for the identified compounds to unravel possible binding interactions and affinities [47,48]. Nineteen compounds were screened against three different protein targets that were extensively incorporated in the process of wound healing by performing molecular docking using the computational program MOE 2019.010. The first protein target is tumor necrosis factor alpha (TNF-*α*) represented by (PDB ID code:2AZ5) co-crystallized with its inhibitor small molecule previously identified by He, M.M., et al. [49]. The second target is the transforming growth factor beta (TGF-*β* receptor type-1), which plays a crucial role in wound healing through regulation of the process of cell differentiation and proliferation rather than its modulatory effect on the immune response (TGF-*β*), represented by protein (PDB ID code:6B8Y) co-crystallized with its new inhibitory ligand the heterobicyclic pyrrolopyrimidine derivative [50]. The final target is IL-1*β* represented by protein (PDB ID code:6Y8M) and co-crystallized with its inhibitory ligand, SX2 (a bromo amido pyridine derivative).

#### 3.5.1. Docking with PDB ID: 2AZ5

**Figure 8:**
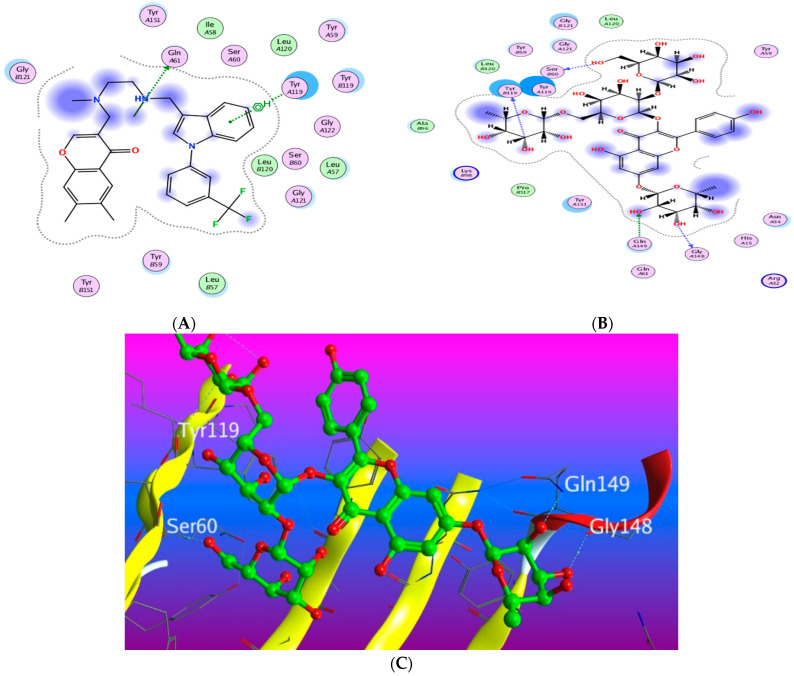
Docking results of compound **14** in the active pocket site of TNF-α (PDB:2AZ5): (**A**) 2D interactions of ligand; (**B**) 2D interactions of compound **14**; (**C**) Docking pose of compound **14**.

The X-ray crystallographic structure of (TNF-*α*) complexed with its ligand was obtained from the Protein Data Bank (http://www.rcsb.org/pdb/, code 2AZ5 (accessed on 10 April 2022). The co-crystallized ligand was found to be bound within a shallow pocket and contacting some amino acid residues from each subunit of the TNF-α dimer. The contact residues composed of 16 amino acids including seven from chain A and nine from chain B (among them six tyrosine residues). The inhibitor acts by binding to the active trimer form of the cytokine and activating the dissociation of this trimer to the inactive dimer and stabilizing it [49]. To validate our study, the ligand was re-docked with the active pocket. Ligand showed interactions with receptor through hydrogen bonds with Gln 61 as H-donor and with Tyr 119 as pi-H interaction (Figure 8). The docking algorithm was able to predict the co-crystalized ligand pose with least RMSD with energy score of −6.923 kcal/mol. 

The dock score of the 19 compounds against 2AZ5 is summarized in (Supplementary Table S2). Docking results revealed that only compound **15** achieved a docking score of −7.544 kcal/mol better than the energy score of the co-crystallized ligand, with more hydrogen bond interactions than the isolated ligand as it showed three hydrogen bonds with Ser 60, Tyr 119, and Gly 148 through the hydroxyl groups of the glucopyranosyl and rhamnopyranosyl moieties in addition to one more hydrogen bond between the oxygen atom in the 7-rhamnopyranosyl moiety and Gln 149. The rest of the compounds showed lower affinity towards the protein than the ligand-like compound **8**, which showed one hydrogen bond interaction and another H-pi interaction in addition to compounds **4**, **7**, and **18** which exhibited two hydrogen bond interactions.

#### 3.5.2. Docking with PDB ID:6B8Y

**Figure 9:**
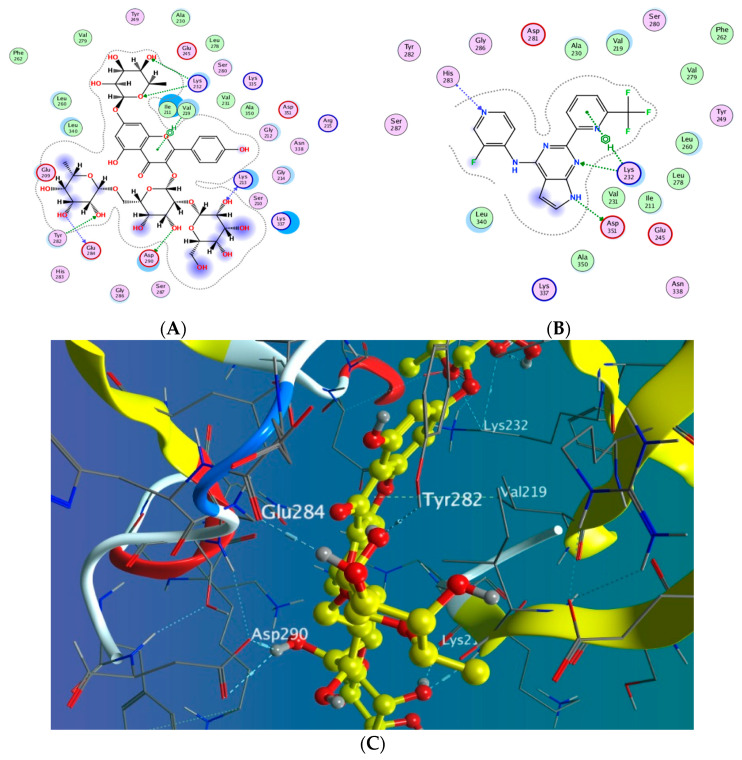
Docking results of compound **14** in the active pocket site of (TGF-β) (6B8Y): (**A**) 2D interactions of ligand; (**B**) 2D interactions of compound **14**; (**C**) 3D Docking pose of compound **14**.

The X-ray crystallographic structure of (TGF-*β*) complexed with its novel pyrrolopyrimidine ligand was obtained from the protein data bank (http://www.rcsb.org/pdb/, code 6B8Y (accessed on 10 April 2022). TGF-*β* is transmembrane serine/threonine kinase that phosphorylate SMAD proteins which will in turn be dimerized and translocated to the nucleus followed by gene transcription [50]. To validate our study, the ligand was re-docked with its active binding site showing hydrogen bond formation between the pyrrole NH and the carbonyl of Asp 351. Two hydrogen bonds are formed with Lys 232 and His 283 through the nitrogen of the pyrimidine ring and the pyridinyl moiety, respectively, in addition to one hydrophobic interaction with Lys 232 (Figure 9). The docking algorithm was able to predict the co-crystalized ligand pose with least RMSD with energy score of −7.555 kcal/mol. 

**Figure 10:**
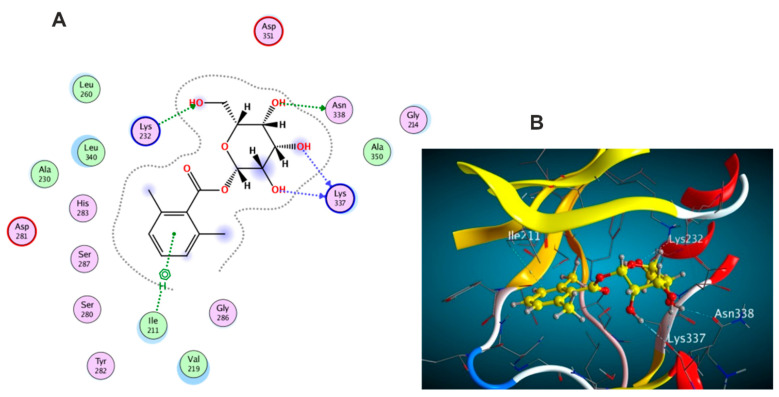
Docking results of compound **1** in the active pocket site of (TGF-β) (6B8Y): (**A**) 2D interactions of compound **1** (**B**) 3D Docking pose of compound **1**.

**Figure 11:**
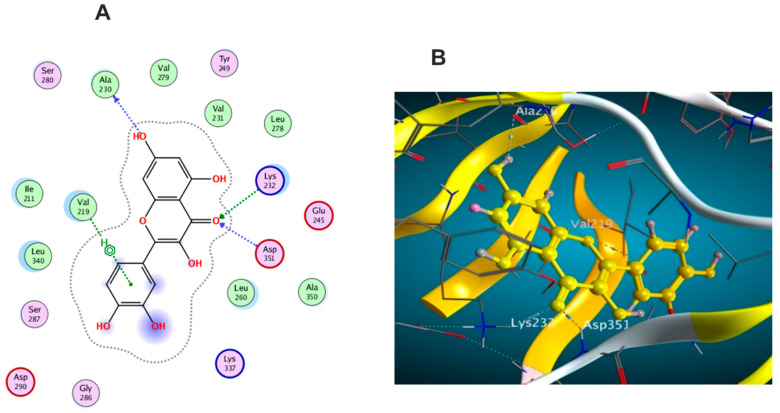
Docking results of compound **3** in the active pocket site of (TGF-β) (6B8Y): (**A**) 2D interactions of compound **3** (**B**) 3D Docking pose of compound **3**.

**Figure 12:**
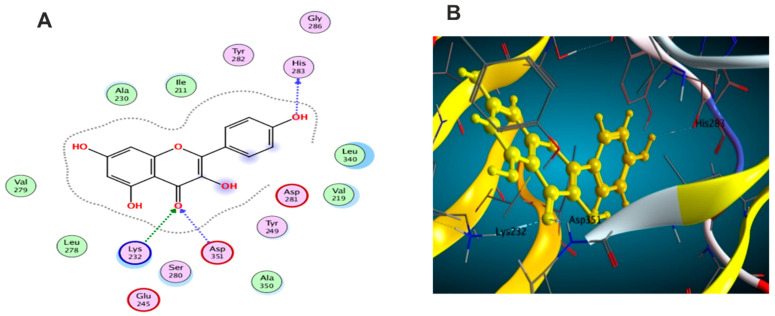
Docking results of compound **4** in the active pocket site of (TGF-β) (6B8Y): (**A**) 2D interactions of compound **4** (**B**) 3D Docking pose of compound **4**.

**Figure 13:**
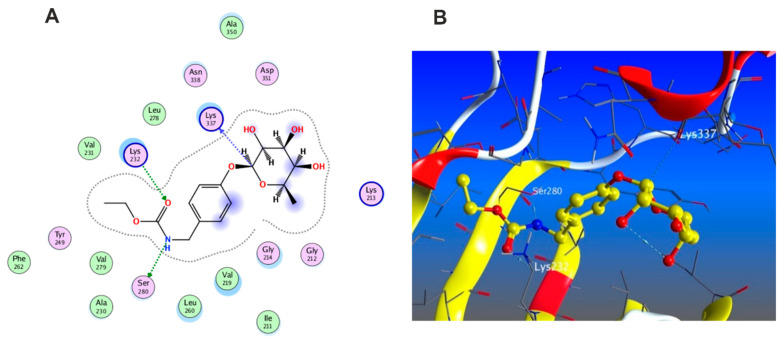
Docking results of compound **16** in the active pocket site of (TGF-β) (6B8Y): (**A**) 2D interactions of compound **16** (**B**) 3D Docking pose of compound **16**.

**Figure 14:**
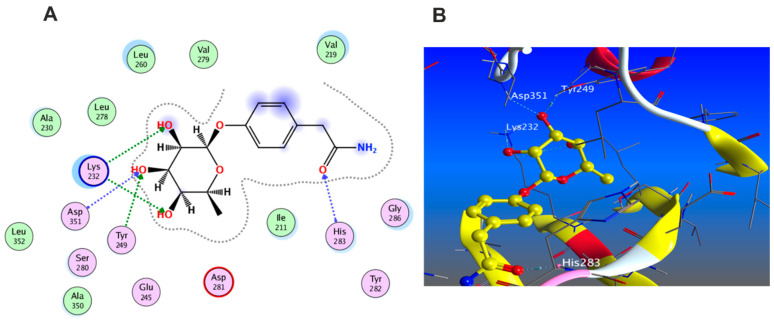
Docking results of compound **18** in the active pocket site of (TGF-β) (6B8Y): (**A**) 2D interactions of compound **18** (**B**) 3D Docking pose of compound **18**.

The docking results of the 19 compounds against (6B8Y) are summarized in Supplementary Table S3. Representative compounds with potential affinity were presented in Figure 10, Figure 11, Figure 12, Figure 13 and Figure 14. The docking results revealed that compound **14** showed the highest affinity towards the protein (6B8Y) as it recorded the lowest energy score among the screened compounds and also much lower than co-crystalized ligand with score of −10.186 kcal/mol. The binding involved seven hydrogen bond interactions, of which three form H-donor with Lys 213, Asp 290, and Glu 284 and the other four form H-acceptor with Lys 213, Tyr 282, and Lys 232 besides one pi-H interaction between the pyrone moiety and Val 219 residue. Compound **16** similarly showed better energy score than ligand −7.654 kcal/mol) forming three hydrogen bonds with the docked receptor, of which one forms H-acceptor with Lys 232 (resembling that formed by the co-crystalized ligand) and another two as H-donor with Ser 280 and Lys 337.

Notably, compounds **1** and **3** achieved nearly equal binding energy scores as the ligand possessed. Meanwhile, compound **18** recorded an energy score of −6.755 kcal/mol although it showed interaction pattern that greatly resembled that achieved with the co-crystalized ligand with the same amino acid residues Asp 351, Lys 232, and His 283. The remaining compounds displayed higher binding energies than the inhibitory ligand. 

#### 3.5.3. Docking with PDB ID:6Y8M

**Figure 15:**
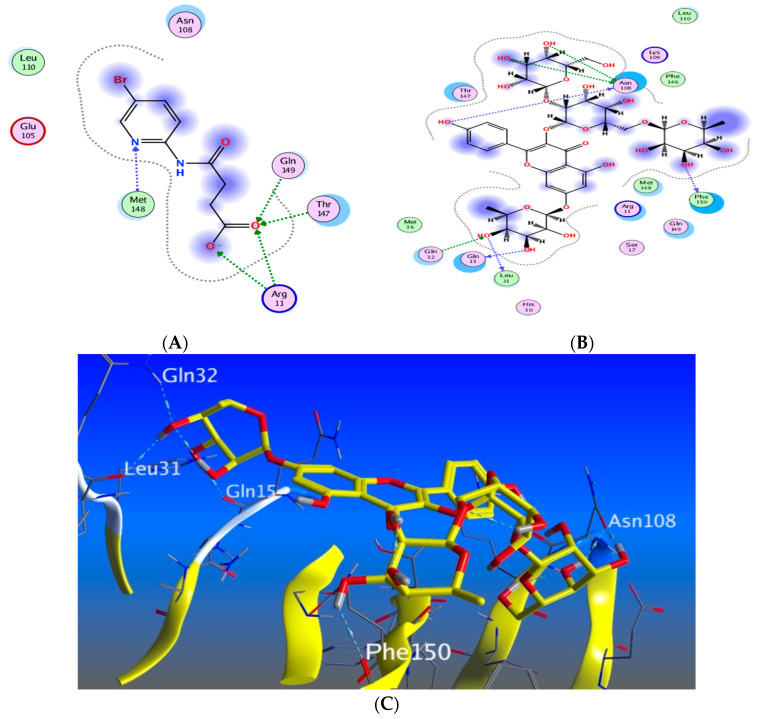
Docking results of compound **14** in the active pocket site of IL-1β (PDB:6Y8M): (**A**) 2D interactions of ligand; (**B**) 2D interactions of compound **14**; (**C**) 3D Docking pose of compound **14**.

**Figure 16:**
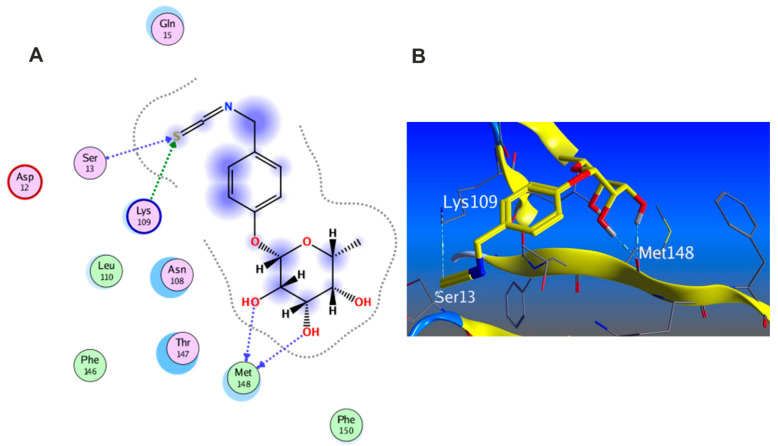
Docking results of compound **15** in the active pocket site of IL-1β (PDB:6Y8M): (**A**) 2D interactions of compound **15** (**B**) 3D Docking pose of compound **15**.

**Figure 17:**
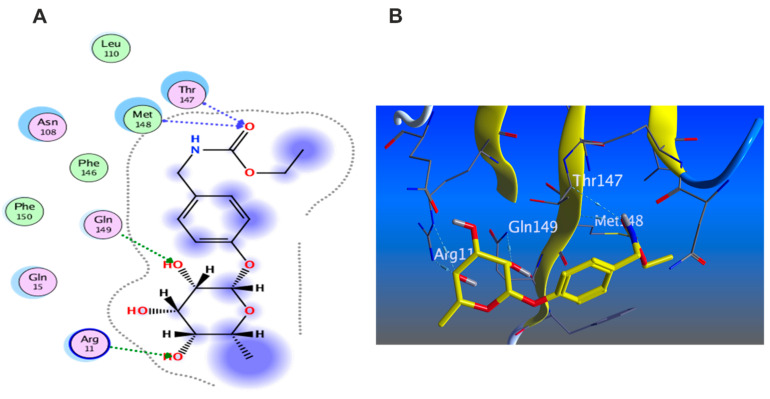
Docking results of compound **16** in the active pocket site of IL-1β (PDB:6Y8M): (**A**) 2D interactions of compound **16** (**B**) 3D Docking pose of compound **16**.

**Figure 18:**
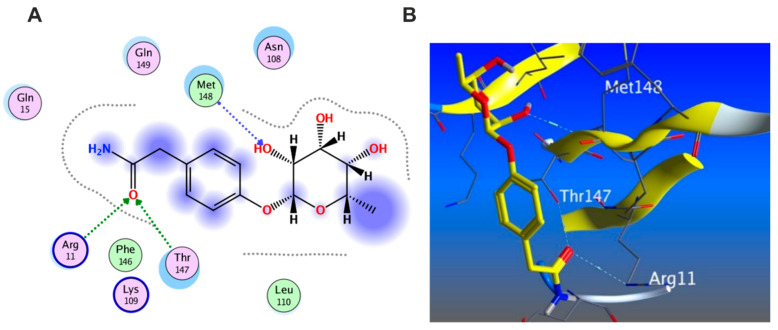
Docking results of compound **18** in the active pocket site of IL-1β (PDB:6Y8M): (**A**) 2D interactions of compound **18** (**B**) 3D Docking pose of compound **18**.

The third target is the cytokine IL-1β represented by protein (PDB ID code:6Y8M) co-crystallized with its inhibitory ligand, SX2. The X-ray crystallographic structure obtained from the protein data bank, upon validation of the docking method, the ligand possessed an energy score of −4.8193 kcal/mol with RMSD 1.1513, showing five hydrogen bond interactions at the binding site with Met 148, Arg 11, Thr 147, and Gln 149 (all as hydrogen bond acceptors). The docking results of the investigated compounds are listed in Supplementary Table S4. Representative compounds with potential activity were presented in Figure 15, Figure 16, Figure 17 and Figure 18. Compound **14** recorded outstanding results as it showed higher affinity to the docked receptor with energy score −7.432 kcal/mol much lower than the one achieved by the ligand −4.8193 kcal/mol. Moreover, it showed more hydrogen bond interactions than the ligand as it displayed seven hydrogen bond interactions with amino acid residues Asn108, Phe105, Leu31, and Gln15 as H-donor and with Gln32 as H-acceptor.

Remarkably, compounds **1**, **15**, **16**, and **18** achieved an energy score of compound-receptor complex lower than the co-crystallized ligand with scores of −5.023, −5.352, −5.541 and −5.118, respectively. Compound **16** showed similar hydrogen bond interactions as the ligand with the same amino acids Met 148, Arg 11, Thr 147, and Gln 149 (Figure 15). Some compounds which recorded comparable energy score results displayed good interactions that greatly resemble the ligand interactions; this was obvious in compounds **2**, **7**, and **8**, while other compounds showed higher energy score than the ligand.

#### 3.5.4. Wound Healing Network Design

Here, we applied a virtual screening-based approach with the purpose of prediction the wound healing capability of *M. oleifera* extracts and their phytoconstituents that may be incorporated with their biological activity

##### Collection of Potential Targets for Wound Healing

Since our aim is to build a PPI network tailored to wound healing, the proposed approach starts with the selection of a list of proteins known to have a role in the wound healing process. These proteins were collected from the following four databases: Gene Cards (https://www.genecards.org/) (accessed on 20 May 2022) [51], the Therapeutic Target Database (TTD, http://db.idrblab.net/ttd/) (accessed on 20 May 2022) [52], the Comparative Toxicogenomic Database (CTD, http:// ctdbase.org/) (accessed on 20 May 2022) and the Drug Bank database (https://www.drugbank.ca/) (accessed on 20 May 2022) [53]. The words ‘‘Wound infection”, ‘‘Surgical wound dehiscence”, and ‘‘Surgical wound infection” were used as keywords to retrieve associated targets and the species were limited as “Homo sapiens”. The analysis of these data with the literature allowed us to extract a list of the most significantly proteins that have been considered the suggested target proteins [54,55].

##### Network Construction

**Figure 19:**
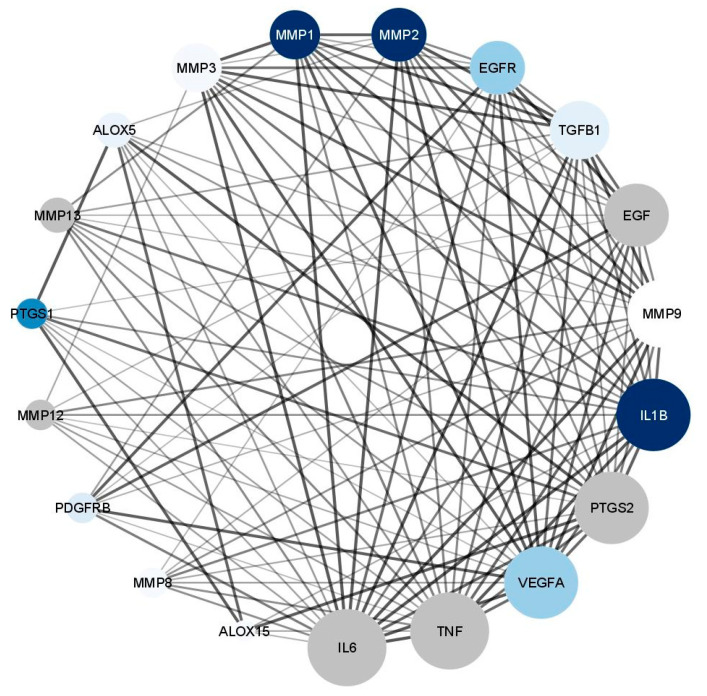
Network nodes represent suggested protein targets, and the edges represent protein–protein interactions. The size of nodes signifies the connectivity of each protein; the higher the node size the higher its connectivity to other nodes; the color fill of the nodes represents the skin tissue expression as the more intense the color the greater the expression.

**Figure 20:**
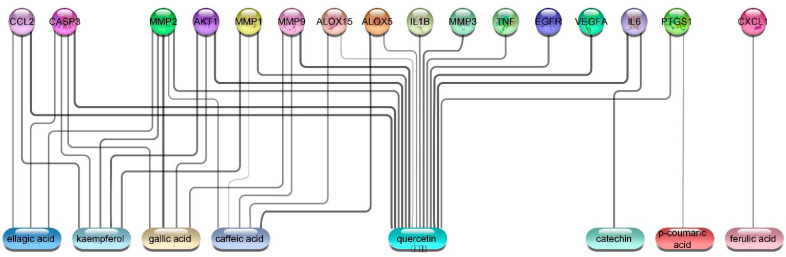
Compound–target network of eight isolated compounds with the potential target proteins, the edges represent a weighted compound–protein connection as the thickness of the edge related to the degree of connectivity, this figure were suggested from the STRING database.

We constructed two networks (Figure 19 and Figure 20). The Protein–Protein Interaction (PPI) Network which displayed the interactions between proteins related to wound healing. The suggested proteins that were expected to be involved in wound healing process were submitted to the STRING application (https://string-db.org/) (accessed on 2 June 2022) [56] for protein–protein interaction (PPI) analysis, selecting “Homo sapiens” as the type of species. The confidence score was set to 0.4 on the default settings for the rest of the parameters to achieve the PPI network. The constructed was network created by Cytoscape 3.9.1 (https://www.cytoscape.org/) (accessed on 5 June 2022) [57], a software package for visualizing and analyzing networks. It is considered a weighted network where the edge weights correspond to the STRING confidence score associated with the PPI (edge). Utilizing the network analyzer tool in Cytoscape, we observed that the network comprised of 19 nodes, 119 edges, and the average number of neighbors was 12.5. The topological parameters such as node degree, betweenness, closeness and the strength of skin tissue expression of each protein are summarized in Table 2. The median of the degree, betweenness, and closeness in the network were 13,0.007625272 and 0.782608696, respectively. The network was visualized in a circular layout using “yFiles circular layout” tool in Cytoscape software and was sorted by linking the size of the nodes with the degree of connectivity. Another classification in the form of color intensity of the nodes and their score of skin tissue expression (the main site of action in wound healing process) is included.

The constructed network revealed that, nodes IL6, TNF, VEGFA, PTGS2, IL1B, and MMP9 recorded the highest score regarding the degree of connectivity; and this finding is in great accordance with several previous reports [58]. The classification of the suspected proteins into a range of (0–4) revealed that proteins PTGS1, MMP1, MMP2, and IL1B achieved a highest score while nodes VEGFA, EGFR, TGF*β*1, and PTGS1 recorded moderate ones. 

The second network is the compound–protein Interaction (CPI) Network, which was constructed to reveal the probable correlations between the suggested targets and the selected compounds. In the network, we constructed connections between the isolated phytochemicals and target proteins relevant to wound healing using STRING application for establishing the network and Cytoscape for visualization [59]. The network revealed that eight compounds exhibited connections with the targeted proteins; among them quercetin, caffeic acid, and kaempferol which showed the highest connectivity with the putative proteins (Figure 20). Additional investigations were performed utilizing the data collected from the two networks to understand the possible molecular mechanisms of the most active compounds quercetin, caffeic acid, and kaempferol, taking into consideration the following parameters like the degree of connectivity of the protein, the skin tissue expression and the compound connectivity to the proteins; accordingly, kaempferol was selected to be tested for MMP2 while caffeic acid was tested with MMP1 and quercetin was tested with IL6.

#### 3.5.5. In Silico Molecular Docking 

**Figure 21:**
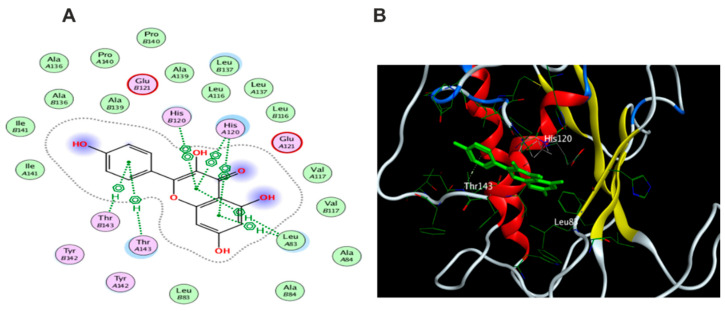
(**A**) 2D interaction of kaempferol with amino acid residues (**B**) 3D pose of kaempferol in the active pocket site MMP2 (PDB ID code:1HOV).

**Figure 22:**
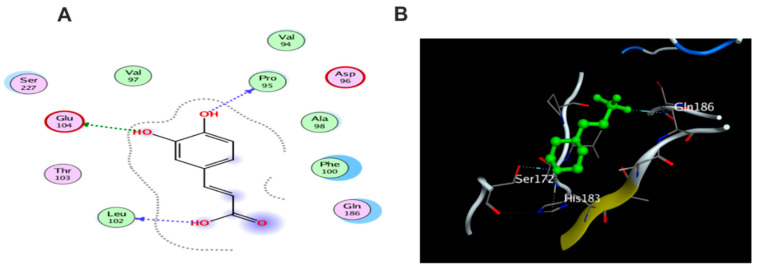
(**A**)2D interaction of caffeic acid with amino acid residues (**B**) 3D pose in the active pocket site MMP1 (PDB ID code:1SU3).

**Figure 23:**
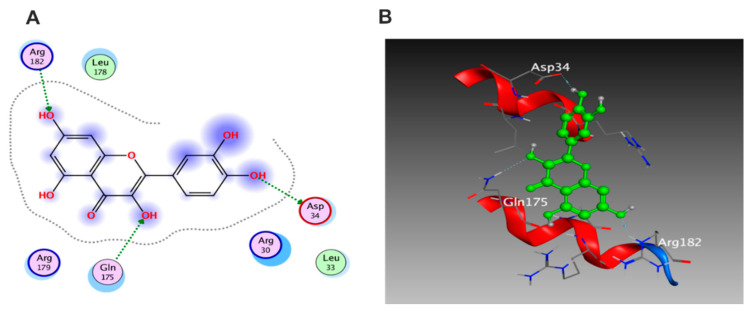
(**A**)2D interaction of quercetin with amino acid residues (**B**) 3D pose in the active pocket site IL6 (PDB ID code:1ALU).

Molecular docking was carried out for the aforementioned three compounds and their related proteins (Figure 21, Figure 22 and Figure 23). Quercetin was docked with the cytokine IL-6 represented by protein (PDB ID code:1ALU) co-crystallized with its inhibitory ligand, TLA (Figure 23). The ligand possessed an energy score of −4.161 kcal/mol with RMSD 2.06 while quercetin showed higher affinity to the docked receptor with energy score −4.486 kcal/mol lower than the one achieved by the ligand. Similarly, caffeic acid achieved a lower energy score when docked with MMP1 protein (PDB ID code:1SU3) than the co-crystallized ligand, EPE, (Figure 22) as it showed ΔG −4.99 kcal/mol and the ligand recorded ΔG −4.22 kcal/mol. The third candidate, kaempferol, docked with the selected protein MMP2 represented in (PDB ID code:1HOV) (Figure 21) and it showed good affinity towards the protein with energy score of −7.028 kcal/mol and RMSD 0.738 compared to the co-crystallized ligand I52 which displayed ΔG −9.018 kcal/mol and RMSD 2.096.

### 3.6. The Effects of the Major Metabolites in M. oleifera Seed Extract on the Inhibition of the Proinflammatory Cytokine Interleukin-6

The flavones quercetin and kaempferol as well as the cinnamic acid derivative, caffeic acid, were assessed for their ability to reduce the proinflammatory cytokine, IL-6. Lipopolysaccharides, a major component in the cell wall of Gram (−ve) bacteria and a potent inducer for an acute inflammatory response, was used as a positive control. While LPS stimulate the release of various cytokines, among them IL-6, kaempferol displayed major suppression of IL-6 by ca. 76% (Table 3); however, quercetin and caffeic acid displayed similar inhibition of IL-6 by ca. 56% and 53%, respectively.

### 3.7. The Effects of the Major Metabolites in Moringa oleifera Seed Extract on the Inhibition of the Endopeptidases, Matrix Metalloproteinases (MMP) 1 and 2

Although matrix metalloproteinases are involved in many critical biological processes like wound healing, angiogenesis, immunity, and bone remodeling, the uncontrolled and dysregulated activity of MMP are observed in cancer and in several inflammatory diseases such as arthritis. Kaempferol showed the highest inhibition for both subtypes of MMP I and II up to 60% and 67%, respectively (Table 4).

### 3.8. In Silico Drug Likeness

Various physicochemical properties could greatly influence the bioactivity of a given drug as it is closely related to the interactions between the drug and its potentially suspected target. Recently in silico approaches introduce a powerful tool for drug discovery to assess the proposed pharmacokinetics (ADME) of compounds which play a vital role in their pharmacological activities, specially at the early stages of screening for lead compounds [60]. Consequently, the measurement of these parameters is of great value in the selection of an efficient drug candidate. Lipinski and Veber rules are successful tools to perform such screening as Lipinski’s rule of five states that a compound has drug-like activity if at least 3 of the following criteria were achieved. A molecular mass less than 500 Da, a maximum of five hydrogen donors, a maximum of 10 hydrogen bond acceptors, and a partition coefficient between octanol and water (LogP (o/w)) smaller than 5 [61]. According to Veber’s rule, the compound is orally active if it has 10 or less rotatable bonds with a polar surface area (PSA) not less than 140Å [62]. For predicting drug likeness properties, we used Reaxys [63]. The screening of the 19 phytochemicals revealed that all of them complied with Lipinski and Veber rules except for compound **15** that does not obey the two rules, and this might be attributed to the large glycosidic moiety attached to the compound (Figures S1–S19, Supplementary File).

## 4. Conclusions

This is the first study evaluating the wound healing potential of *M. oleifera* seed extract with insights to its chemical profile. The extract showed notable wound healing potential by accelerating wound closure rate, increasing the expression of *TGF-β1*, VEGF, type I collagen, and decreasing the inflammatory markers as well as the relative gene expression of *IL-1β* and *TNF-α*. The seed extract displayed a likewise strong scavenging effect to peroxides and superoxide free radicals. In silico studies of the identified compounds gave a putative prediction to the possible mechanism by which *M. oleifera* seed extract exerts its wound healing effect. Compounds **14** and **16** possessed higher affinities to the screened receptors with binding energy scores better than the ligand. Similarly, compounds **1**, **3**, **15**, and **18** showed good energy scores with enhanced binding interactions. Protein–protein interaction (PPI) and compound–protein interaction (CPI) networks were constructed. Quercetin, caffeic acid, and kaempferol showed the highest connectivities with the putative proteins. In silico drug likeness studies revealed that almost all compounds comply with both Lipinski rule and Veber rule. In vitro studies further supported the in silico data since kaempferol could efficiently supress interleukin-6 as well as MMPs I and II.

## Figures and Tables

**Table 1:** Tentative identification of key metabolites in *Moringa oleifera* seeds extract. Identification of the compounds was based on HR-ESIMS and comparison with the data reported in the literature.

Peak No.	Identified Metabolite	Chemical Structure	Exact Mass	Phytochemical Class	Ref.
1	Moringyne	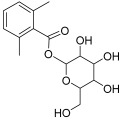	312.12090	Phenolic acid derivative	[21]
2	Catechin	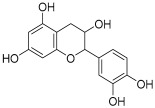	290.07904	Flavan-3-ol	[22]
3	Quercetin	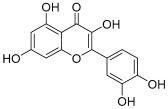	302.042655	Flavonol	[22]
4	Kaempferol	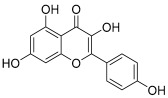	286.04774		[23]
5	Gallic acid	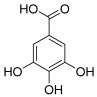	170.021525	Benzoic acid derivative	[24]
6	*p*-Coumaric acid	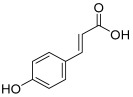	164.047345	Cinnamic acid derivative	[22]
7	Ferulic acid	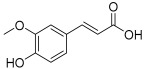	194.058	Cinnamic acid derivative	[22]
8	Caffeic acid	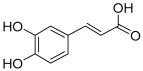	180.04226		[22]
9	Protocatechuic acid	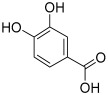	154.02661	Benzoic acid derivative	[22]
10	Cinnamic acid	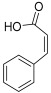	148.05243	Organic acid	[22]
11	Ellagic acid	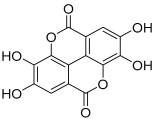	302.00627	Polyphenol	[23]
12	Vanillic acid	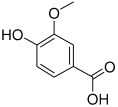	168.04226	Benzoic acid derivative	[22]
13	Benzylamine	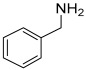	107.073499	Organic amine	[25]
14	Kaempferol 3,7-diglycosides 3-O-[β-D-Glucopyranosyl-(1→2)-[α-L-rhamnopyranosyl-(1→6)]-β-D-glucopyranoside], 7-O-α-L-rhamnopyranoside	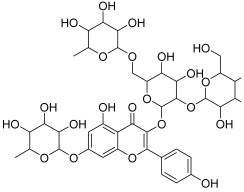	902.26921	Flavonol glycoside	[26]
15	4-Hydroxybenzyl isothiocyanate *O*-α-L-rhamnopyranoside	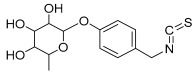	311.082744	Isothiocyanate derivative	[27]
16	(4-Hydroxybenzyl) carbamic acid ester, *O*-α-L-rhamnopyranoside	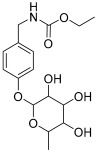	341.147454	Carbamate derivative	[28]
17	Rhamnose; α-L-pyranose-form, Phenolic glycoside	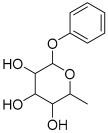	240.0997	Phenolic glycoside	[29]
18	4-Hydroxyphenylacetic acid amide, *O*-α-L-rhamnopyranoside	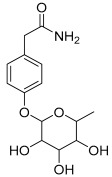	297.121239	Phenolic glycoside	[29]
19	3,4-Dihydro-4,8-dihydroxy-3-methyl-1H-2-benzopyran-1-one; (3*R*,4*S*)-form	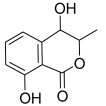	194.05791	Isocoumarin	[30]

**Table 2:** Topological parameters of targets combined with skin tissue expression score.

No	Name	Target	Degree	Betweenness	Closeness	Tissue/Skin
1	Tumor necrosis factor	TNF	18	0.058613445	1	0.72563
2	Interleukin 6	IL6	18	0.058613445	1	0.88975
3	Vascular endothelial growth factor A	VEGFA	17	0.03889667	0.947368421	1.449221
4	Prostaglandin-endoperoxide synthase 2	PTGS2	17	0.045650482	0.947368421	0.745856
5	Interleukin 1B	IL1B	17	0.045650482	0.947368421	4
6	Matrix metallopeptidase 9	MMP9	16	0.027552132	0.9	0.166331
7	Epidermal growth factor	EGF	15	0.021864301	0.857142857	0.698752
8	Transforming growth factor beta 1	TGFβ1	14	0.012044818	0.818181818	0.94118
9	Epidermal growth factor receptor	EGFR	13	0.007625272	0.782608696	1.459392
10	Matrix metallopeptidase 2	MMP2	13	0.009126984	0.782608696	2.805839
11	Matrix metallopeptidase 1	MMP1	12	0.002178649	0.75	4.357237
12	Matrix metallopeptidase 3	MMP3	12	0.003267974	0.75	0.475876
13	Matrix metallopeptidase 13	MMP13	9	0	0.666666667	0.413252
14	Arachidonate 5-Lipoxygenase	ALOX5	9	0.005571117	0.666666667	0.753328
15	platelet-derived growth factor receptor beta	PDGFRβ	8	0	0.642857143	1.185637
16	Matrix metallopeptidase 12	MMP12	8	0	0.642857143	0.658791
17	Matrix metallopeptidase 8	MMP8	8	0	0.642857143	0.44169
18	Prostaglandin-endoperoxide synthase 1	PTGS1	8	0.003213508	0.642857143	2.071426
19	Arachidonate 15-Lipoxygenase	ALOX15	6	0	0.6	0.102662
	Median		13	0.007625272	0.782608696	0.753328

**Table 3:** The effect of Moringa metabolites viz. quercetin, kaempferol, and caffeic acid on the proinflammatory cytokine IL-6.

Metabolite Name	IL-6 Concentration (pg/mL)	Approximate % of IL-6 Relative to LPS
Quercetin	71.51 ± 1.56	43.4%
Kaempferol	39.65 ± 1.19	24%
Caffeic acid	77.41 ± 1.79	47%
LPS (as control)	164.7 ± 13.7	100%

**Table 4:** The effects of Moringa metabolites viz. quercetin, kaempferol, and caffeic acid on matrix metalloproteinases type I and II.

Compound Name	MMP-1 (pg/mL)	Approximate % of MMP-1 Relative to LPS	MMP-2 (pg/mL)	Approximate % of MMP-2 Relative to LPS
Quercetin	2160 ± 30.7	67.8%	170.3 ± 2.49	59.7%
Kaempferol	1276 ± 52.2	40.1%	92.59 ± 3.36	32.4%
Caffeic acid	1926 ± 49.1	60.5%	199.3 ± 1.56	69.9%
LPS (as control)	3183 ± 78.8	100%	285 ± 10.9	100%

## Data Availability

Data is contained within the article or Supplementary Material.

## Supplementary Materials

The following supporting information can be downloaded at: https://www.mdpi.com/article/10.3390/antiox11091743/s1, Figures S1–S19: In silico drug likeness of compounds (**1**–**19**); Table S1: The primer sequences of studied genes. Table S2: Receptor interactions and binding energies of the 19 Compounds and ligand into the active pocket site of TFN-α catalytic domain. Table S3: Receptor interactions and binding energies of the 19 compounds and ligand into the active pocket site of (TGF-β) catalytic domain. Table S4: Receptor interactions and binding energies of the 19 compounds and ligand into the active pocket site of IL-1*β* catalytic domain [18,19,20,22,23].
