# Supplementary material for: Mechanistic Wound Healing and Antioxidant Potential of Moringa oleifera Seeds Extract Supported by Metabolic Profiling, In Silico Network Design, Molecular Docking, and In Vivo Studies
Source: Antioxidants (Basel). 2022 Sep 1;11(9):1743. doi: 10.3390/antiox11091743 (PMC9495458; doi:10.3390/antiox11091743)
Supplement: Supplementary file 1 [file antioxidants-11-01743-s001.zip › antioxidants-1848603-supplementary.pdf]

Supplementary Data

Mechanistic Wound Healing and Antioxidant Potential of *Moringa oleifera* Seeds Extract Supported by Metabolic Profiling, In-Silico Network Design and molecular Docking, and In-Vivo Studies

2. Material and methods:

2.1. Animal Administration and Wound Excision Model:

The rabbits were anesthetized by intraperitoneal (I.P.) injection with ketamine (Alphasam company®, Holland, 50 mg/kg) and xylazine hydrochloride (Alphasam company®, Holland, 10 mg/kg) [1]. After anesthesia, the awareness (alertness) level of rabbits was determined, and shaving was done. The shaving area was back of the animal, in the withers. Anticipation was done by alcohol 70% and povidone-iodine 10% 7 times. The animals were depilated on the paravertebral area before wound creation and a circular excision wound of 6 mm in diameter was created using a biopsy punch [2]. This procedure generates the wound in both the epidermis and the dermis layers. Using experimental rabbits three groups were formed, each group include 8 rabbits. The rabbits of group 1 did not receive any treatment (bare wound) and were used as the negative control. Wounds in group 2 were treated with *M. oleifera seeds extract* (2 mg/wound), while group 3 was treated with MEBO ointment (100 mg/wound) and were used as positive control (Market treatment). The wounded area was covered with a standard surgical dressing while redressing was performed with fresh dressing on 3, 7 and 10 days.

2.2. Wound healing evaluation

For the physical appearance and closure of wounds, photographs of wounded areas were taken by using a digital camera (DSC-W320 Sony; Sony Corp., Tokyo, Japan) on 0, 3, 7, 10, and 14 days posing vertically to middle of wound with a distance of 6 cm. The reduction in wounded area (wound contraction) was used as an indicator of efficacy of the treatment. Thus, the periphery of the excisional wound was outlined after creating the wound with the help of transparent paper. The contraction in wounds was recorded on 3, 7, 10, and 14 days and expressed as a percent of the healed wounded area. The percentage wound contraction was estimated using the formula:

Wound contraction = Area of original wound-Area at nth day/ Area of original wound x 100

Moreover, the day of complete wound healing (epithelialization) of each wound was observed.

2.3. Total RNA extraction

About 50 mg of dorsal skin tissues was homogenized by ultrasonic homogenizer (Sonics-Vibracell, Sonics& Materials Inc., Newtown, USA) in 0.5 ml TRIzol TM reagent (Amresco, Solon, USA). Total RNA was extracted from dorsal skin tissues using TRIzol RNA extraction reagent (Amresco, Solon, USA) following the manufacturer's instructions. The total RNA concentration was determined at A=260 nm and the purity was calculated according to the ratio A260/A280. Samples having a purity ≥ 1.7 were selected for qRT-PCR using GAPDH (Glyceraldehyde-3-phosphate dehydrogenase) as a reference housekeeping gene.

2.4. Real-time qRT-PCR

cDNA synthesis was performed for equal quantities of total RNA in all samples using the RevertAid H Minus First Strand cDNA Synthesis kit (#K1632, Thermo Scientific Fermentas, St. Leon-Ro, Germany) according to the manufacturer's instructions. Real-time PCR was carried out with single-stranded cDNAs. PCR reactions were performed by SYBER Green [#K0251, Thermo Scientific Fermentas St. Leon-Ro, Germany-Maxima SYBER Green qPCR Master Mix (2X)] using StepOne Real-Time PCR Detection System (Applied Biosystems). The set of primers used for Real-Time PCR were mentioned in **Table S1**. Real-time polymerase chain reaction (qRT-PCR) was carried out using 20 µl of RealMOD Green qRT-PCR Mix kit (iNtRON biotechnology) with 0.02 µg RNA per reaction containing 10 pmol of specific primers, for 30 cycles of 95°C for 10 sec. and 60°C for 1 min. Comparative Ct (threshold cycle) method was used to determine the relative amounts of the products. The relative expression was calculated using the formula $2^{(-\Delta\Delta Ct)}$ [3]. They were scaled relative to controls where control samples were set at a value of 1.

Table S1. The primer sequences of studied genes.

Gene name	GenBank accession		
IL- β1	NC_013670.1	Forward	5'-AGCTTCTCCAGAGCCACAAC-3'
		Reverse	5'-CCTGACTACCCTCACGCACC-3'
GAPDH	NC_013676.1	Forward	5'-GTCAAGGCTGAGAACGGGAA-3'
		Reverse	5'-ACAAGAGAGTTGGCTGGGTG-3'
TGF-β1	NC_013672.1	Forward	5'-GACTGTGCGTTTTGGGTTCC-3'
		Reverse	5'-CCTGGGCTCCTCCTAGAGTT-3'
TNF-α	NC_013680.1	Forward	5'-GAGAACCCCACGGCTAGATG-3'
		Reverse	5'-TTCTCCAACTGGAAGACGCC-3'

3-. Histopathological analysis

Rabbits were anesthetized on 7th and 14th days and the wounded area with a periphery of about 5 mm of ambient un-wounded skin biopsy was taken. These skin tissues were fixed a 10% formalin solution for 2-3 days followed by tissue processing and embedding in paraffin. Thin tissue sections of 5 µm thickness were made using Microtome and stained with hematoxylin and eosin (H&E). The stained sections were observed using a light microscope fitted with a camera about neovascularization, epidermis, scar, and granulation tissues, and images were taken.

4- *In-Vitro* Antioxidant Activity

4.1. Hydrogen Peroxide Scavenging Activity

The reaction with a defined amount of exogenously provided H_2O_2 was used to determine the hydrogen peroxide (H_2O_2) scavenging activity that reflects the anti-oxidative capacity of *M. oleifera seeds extract*. Colorimetric analysis was used to estimate the residual H_2O_2 [4]. In brief, 20 µl of the extract was mixed with 500 µl of H_2O_2 and incubated at 37ºC for 10 minutes. After that, 500 µl of enzyme/3, 5-dichloro-2-hydroxyl-benzenesulfonate solution was added and incubated at 37 ºC for 5 minutes. Colorimetrically, the intensity of the colored product was measured at 510 nm. Ascorbic acid was used as a positive control. By comparing the percentages of H_2O_2 scavenging activity was determined by comparing the results of the *M. oleifera seeds extract* with those of the control using the following formula:

$$\text{scavenging activity} = \frac{\text{A control} - \text{A sample}}{\text{A control}} \times 100$$

IC_{50} of each sample was calculated after performing the assay at four different concentrations using Graph pad prism 7 software.

4.2. Superoxide Radical Scavenging Activity

The superoxide anion scavenging activity was measured as described by Srinivasan R. *et al.* [5]. The superoxide anion radicals were formed in a Tris-HCl buffer (16 mM, pH 8.0) containing 90 µl of NBT (0.3 mM), 90 µl of NADH (0.936 mM), 0.1 ml of *M. oleifera seeds extract* (125, 250, 500, and 1000 µg/mL), and 0.8 mL Tris- HCl buffer (16 mM, PH 8.0). The reaction was initiated by adding 0.1 ml PMS solution (0.12 mM) to the mixture, which was then incubated at 25˚C for 5 minutes, and measured at 560 nm, the absorbance was measured. Ascorbic acid was selected as a reference. The percentage inhibition was obtained by comparing the test findings to those of the control using the formula below:

$$\text{Superoxide scavenging activity} = \frac{\text{A control} - \text{A sample}}{\text{A control}} \times 100$$

IC_{50} was calculated using Graph pad prism 7 software by performing the test at four different concentrations.

5-. Statistical analysis

The statistical analysis was performed using GraphPad Prism (LaJolla, CA). Shapiro Wilk test for normality of variance and then nonlinear fit of normalized variables were performed. Leven's test for homogeneity of variance was performed. Finally Two-way ANOVA was performed. The results are represented as mean ± standard deviation (SD). Two-way ANOVA was applied to determine whether the results have significant variations and a P-value ≤ 0.05 was considered significant.

Results:

1. -Docking study

Table S2. Receptor interactions and binding energies of the 19 Compounds and ligand into the active pocket site of TFN-α catalytic domain.

Compound No.	S_a (kcal/mole)	RMSD_Refine$_b$	Amino acid/ bond	Distance (A˚)	E (Kcal/mol)
1	-5.513	1.013	Tyr 151/H-acceptor<br>Tyr 59 / H-pi	3.00<br>3.75	-1.20<br>-0.80
2	-4.613	1.491	Tyr 119/H-pi	4.12	-0.50
3	-5.591	2.036	Tyr 151/H-acceptor<br>Tyr 59 / pi-pi	3.04<br>3.74	-3.00<br>0.00
4	-5.549	1.088	Ser 60 /H-donor<br>Gly 122/ H-donor	2.96<br>3.15	-1.50<br>-0.60
5	-4.326	0.884	Gly 121/H-donor	3.13	-1.20
6	-4.918	1.513	Ser 60 /H-donor	2.90	-1.20
7	-5.259	1.251	Tyr 151/H-acceptor<br>Ser 60 /H-donor	3.06<br>2.68	-1.02<br>-1.82
8	-4.857	1.174	Ser 60 /H-donor<br>Tyr 59 / pi-H	3.04<br>3.76	-0.70<br>-0.50
9	-4.181	2.051	Ser 60 /H-donor	2.85	-0.80
10	-4.277	1.542	Ser 60 /H-donor	2.92	-1.50
11	-5.408	1.345	Gly 121/pi-H	4.18	-1.00
12	-4.707	1.638	Gly 121/pi-H	3.61	-0.50
13	-4.024	1.898	-------------------	---------------	--------------
14	-7.544	1.533	Ser 60 /H-donor	2.83	-1.5

			Tyr 119/ H-donor	3.45	-0.5
			Gly 148/ H-donor	3.02	-1.2
			Gln 149/ H-acceptor	3.06	-1.4
15	-5.731	1.587	Tyr 151/ H-acceptor	2.92	-1.50
16	-5.904	1.971	Tyr 119/ H-donor	2.87	-1.30
17	-4.968	1.504	GLY 121 /H-donor	3.02	-1.30
18	-5.497	1.341	Gly 121/ H-donor	2.86	-0.6
			Ser 60 /H-donor	3.00	-0.7
19	-4.646	1.738	-------------------	---------------	--------------
#	-6.923	1.718	Gln 61 / H-donor	3.27	-0.70
			Tyr 119/ pi-H	4.12	-0.60

[a] S: the score of a compound placement inside the protein binding pocket.
[b] RMSD_Refine: the root-mean-squared-deviation (RMSD) between the predicted pose and those of the crystal one (after and before refinement process, respectively).

Table S3. Receptor interactions and binding energies of the 19 compounds and ligand into the active pocket site of (TGF-β) catalytic domain.

Compound No.	S[a] (kcal/mole)	RMSD_Refine[b]	Amino acid bond	Distance A°	E (Kcal/mol)
1	-7.205	1.084	Asn 338/H-donor	2.95	-1.40
			Lys337/H-donor	2.97	-1.00
			Lys337/H-donor	2.99	-3.00
			Lys332/H-acceptor	3.17	-0.90
			Ile 211/ pi-H	4.22	-0.60
2	-6.514	1.328	Asp 281 /H-donor	3.06	-1.40
			Glu 245/ H-donor	2.62	-1.70
			His 283 / H-acceptor	3.02	-0.60
			Asp 281 /H-donor	2.87	-1.00
3	-7.05	1.497	Ala 230 / H-donor	3.05	-0.60
			Lys332/H-acceptor	3.00	-3.10
			Asp351/H-acceptor	2.89	-2.30
			Val 219 / pi-H	4.43	-0.70
4	-6.934	1.467	His 283 / H-donor	2.88	-0.60
			Lys332/H-acceptor	3.23	-3.30
			Asp351/H-acceptor	3.30	-0.60
5	-5.402	1.12	------------------------	--------	----------------
6	-5.21	1.81	Asp 281 /H-donor	3.40	-1.20
			His 283 / H-acceptor	3.22	-0.50
7	-5.9	1.541	Asp 281 /H-donor	3.31	-1.50
8	-5.41	1.503	Asp 281 /H-donor	3.40	-1.00
			Lys332/H-acceptor	3.02	-3.30
			Val 219 / pi-H	4.19	-0.70
9	-5.451	0.501	Lys332/H-acceptor	3.22	-0.60
			Tyr 249/H-acceptor	3.01	-1.20
			Asp351/H-acceptor	2.83	-2.80
			Lys332/pi-H	4.06	-0.60
10	-5.247	1.169	Asp351/H-acceptor	3.03	-1.00
			Asp351/H-acceptor	3.21	-0.60
11	-6.163	1.591	Asp 281 /H-donor	3.03	-2.30
			His 283 / H-donor	3.12	-0.70
			Val 219 / pi-H	4.27	-0.50
			Val 219 / pi-H	3.95	-0.70

12	-5.74	0.54	Glu 245/ H-donor	2.78	-2.50
			Asp351/H-acceptor	2.86	-0.60
13	-4.643	1.081	Tyr 249/H-acceptor	3.01	-1.10
			Asp351/H-acceptor	3.24	-2.00
			Lys 232/ pi-H	3.83	-0.80
14	-10.186	1.982	Lys 213/H-donor	3.11	-1.30
			Asp 290/H-donor	3.22	-0.60
			Glu 284/ H-donor	2.98	-2.20
			Lys 213/H-acceptor	2.89	-1.90
			Tyr 282/H-acceptor	3.06	-0.70
			Lys 232/H-acceptor	3.32	-0.50
			Lys 232/H-acceptor	3.15	-0.60
			Val 219 / pi-H	4.22	-0.50
15	-6.595	1.407	Asp 290/H-donor	3.36	-0.50
			Val 219 / pi-H	3.81	-0.50
16	-7.654	1.169	Ser 280 /H-donor	2.91	-2.10
			Lys 337/H-donor	3.10	-0.50
			Lys 232/ H-acceptor	3.36	-0.90
17	-6.079	1.423	Lys 232/ H-acceptor	2.95	-3.50
			Asp351/H-acceptor	3.19	-1.10
			Lys 232/ H-acceptor	3.29	-0.60
18	-6.755	1.689	His 283 /H-acceptor	3.18	-2.10
			Lys 232/ H-acceptor	2.91	-0.60
			Tyr 249/H-acceptor	2.84	-1.80
			Asp351/H-acceptor	3.05	-0.50
			Lys 232/ H-acceptor	2.97	-0.70
19	-6.087	0.618	Lys 232 /H-acceptor	2.98	-1.80
			Tyr 249/H-acceptor	3.17	-0.70
			Asp 351/H-acceptor	2.96	-1.50
#	-7.555	1.535	Asp 351/ H-donor	2.92	-4.90
			Lys 232/ H-acceptor	3.40	-0.70
			His 283/ H-acceptor	3.10	-2.30
			Lys 232/ pi-H	4.21	-0.50

[a] S: the score of a compound placement inside the protein binding pocket.
[b] RMSD_Refine: the root-mean-squared-deviation (RMSD) between the predicted pose and those of the crystal one (after and before refinement process, respectively).

Table S4. Receptor interactions and binding energies of the 19 compounds and ligand into the active pocket site of IL-1β catalytic domain.

Compound No.	S[a] (kcal/mole)	RMSD_Refine[b]	Amino acid bond	Distance (Å)	E (Kcal /mol)
1	-5.023	1.26	Arg 11 /H-acceptor	3.20	-1.40
			Thr 147/ H-acceptor	3.01	-1.00
2	-4.732	1.374	Met 148/ H-donor	2.90	-2.90
			Thr 147/ H-acceptor	2.93	-1.20
			Gln 149/ H-acceptor	3.12	-1.50
			Arg 11 /pi-cation	4.42	-0.60
			Gln 149/ pi-H	4.57	-0.70
3	-4.681	1.47	Asn 108/ H-donor	3.01	-0.60
			Gln 149/ pi-H	4.23	-0.50
4	-4.707	1.368	Met 148/ pi-H	4.40	-0.50
			Gln 149/ pi-H	4.53	-0.50

5	-3.999	1.004	Asn 108/ H-donor<br>Met 148/ pi-H	2.98<br>4.63	-0.50<br>-0.50
6	-3.945	1.195	Asn 108/ H-donor<br>Arg 11/ H-acceptor<br>Arg 11/ H-acceptor<br>Gln 149/ H-acceptor	3.07<br>3.18<br>3.40<br>3.14	-1.00<br>-2.90<br>-1.60<br>-0.80
7	-4.327	1.208	Asn 108/ H-donor<br>Arg 11/ H-acceptor<br>Arg 11/ H-acceptor<br>Gln 149/ H-acceptor	3.01<br>3.28<br>3.42<br>3.22	-0.80<br>-2.20<br>-1.60<br>-0.70
8	-4.107	1.108	Asn 108/ H-donor<br>Arg 11/ H-acceptor<br>Arg 11/ H-acceptor<br>Gln 149/ H-acceptor<br>Met 148 /H-donor	3.18<br>3.25<br>3.10<br>3.35<br>3.25	-1.50<br>-0.60<br>-3.30<br>-1.90<br>-0.50
9	-3.868	1.393	Met 148 /H-donor<br>Asn 108/ H-donor	2.90<br>2.84	-2.30<br>-3.80
10	-3.94	1.143	--------------------	--------	---------
11	-4.077	2.451	--------------------	--------	---------
12	-4.25	1.524	Met 148 /H-donor<br>Asn 108/ H-donor<br>Met 148/ pi-H	3.05<br>2.83<br>4.49	-0.50<br>-3.30<br>-0.60
13	-3.988	1.012	Met 148 /H-acceptor	3.51	-1.00
14	-7.432	1.546	Asn 108/ H-donor<br>Asn 108/ H-donor<br>Asn 108/ H-donor<br>Phe 105/H-donor<br>Leu 31/H-donor<br>Gln 15 /H-donor<br>Gln 32/ H-acceptor	3.07<br>2.95<br>2.77<br>3.05<br>3.13<br>3.04<br>2.96	-1.10<br>-2.30<br>-1.40<br>-1.20<br>-0.70<br>-1.80<br>-2.10
15	-5.352	1.135	Met 148 /H-donor<br>Met 148 /H-donor<br>Ser 13/ H-acceptor<br>Lys 109 / H-acceptor	3.02<br>2.89<br>3.94<br>3.62	-1.10<br>-2.50<br>-0.70<br>-1.40
16	-5.541	1.926	Thr 147/ H-acceptor<br>Met 148 /H-donor<br>Arg 11/ H-acceptor<br>Arg 11/ H-acceptor<br>Gln 149/ H-acceptor	3.40<br>2.96<br>3.23<br>2.96<br>2.84	-0.80<br>-1.50<br>-1.90<br>-4.10<br>-2.50
17	-4.619	1.404	Asn 108/ H-donor<br>Thr 147 /H-acceptor<br>Thr 147 /pi-H	3.18<br>3.43<br>3.65	-0.50<br>-0.70<br>-0.60
18	-5.118	1.293	Arg 11/ H-acceptor<br>Thr 147/H-acceptor<br>Met 148/ H-acceptor	3.41<br>3.25<br>2.96	-0.70<br>-0.90<br>-2.60
19	-4.203	1.374	Thr 147/H-acceptor<br>Met 148/ H-acceptor	3.38<br>3.52	-1.10<br>-1.00
#	-4.8193	1.1513	Met 148/ H-acceptor<br>Arg 11/ H-acceptor<br>Arg 11/ H-acceptor<br>Thr 147/ H-acceptor<br>Gln 149/ H-acceptor	3.57<br>3.41<br>3.62<br>2.83<br>3.06	-0.60<br>-2.30<br>-0.70<br>-3.50<br>-1.40

[a] S: The score of a compound placement inside the protein binding pocket.

[b] RMSD_Refine: the root-mean-squared-deviation (RMSD) between the predicted pose and those of the crystal one (after and before refinement process, respectively).

In silico **drug likeness**

Druglikeness

Lipinski rules component	
Molecular Weight	312.32
logP	1.205
HBA	7
HBD	4
Matching Lipinski Rules	4

Veber rules component	
Polar Surface Area (PSA)	116.45
Rotatable Bond (RotB)	4
Matching Veber Rules	2

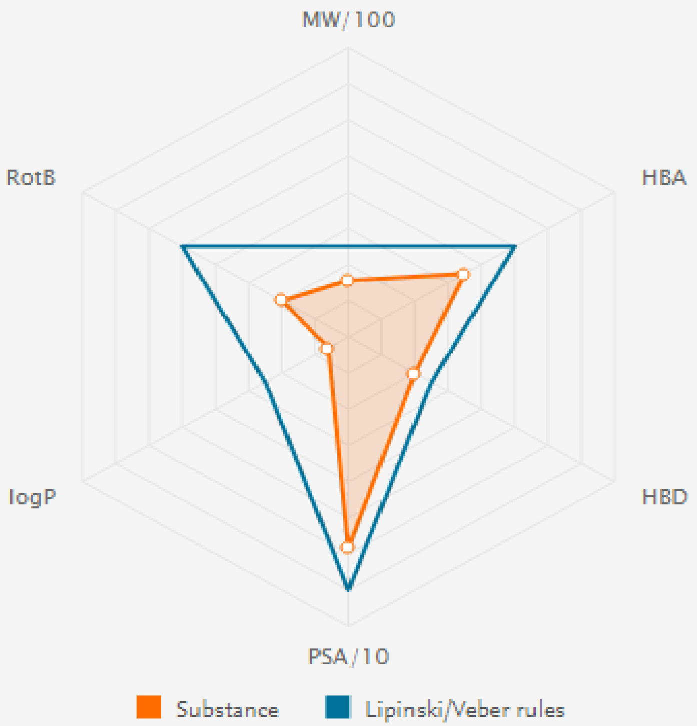

Figure S1. ***In silico*** **drug likeness of** compound **1.**

Druglikeness

Lipinski rules component

Molecular Weight	290.273
logP	1.461
HBA	1
HBD	5
Matching Lipinski Rules	4

Veber rules component

Polar Surface Area (PSA)	110.38
Rotatable Bond (RotB)	1
Matching Veber Rules	2

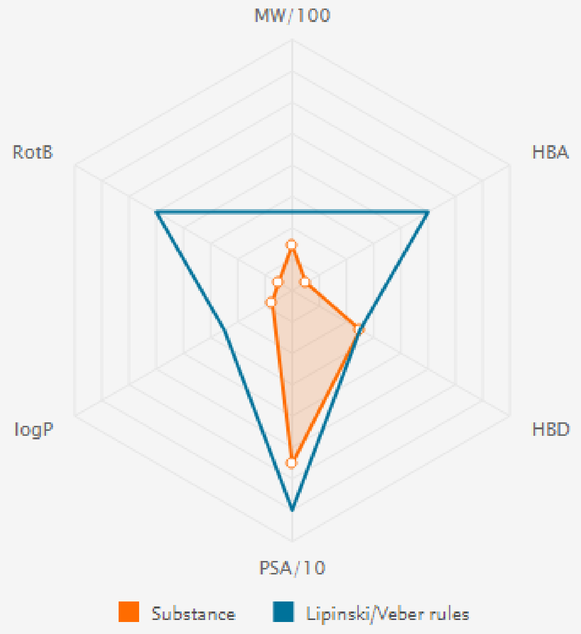

Figure S2. ***In silico*** **drug likeness of** compound **2.**

Druglikeness

Lipinski rules component

Molecular Weight	302.24
logP	2.517
HBA	2
HBD	5
Matching Lipinski Rules	4

Veber rules component

Polar Surface Area (PSA)	127.45
Rotatable Bond (RotB)	1
Matching Veber Rules	2

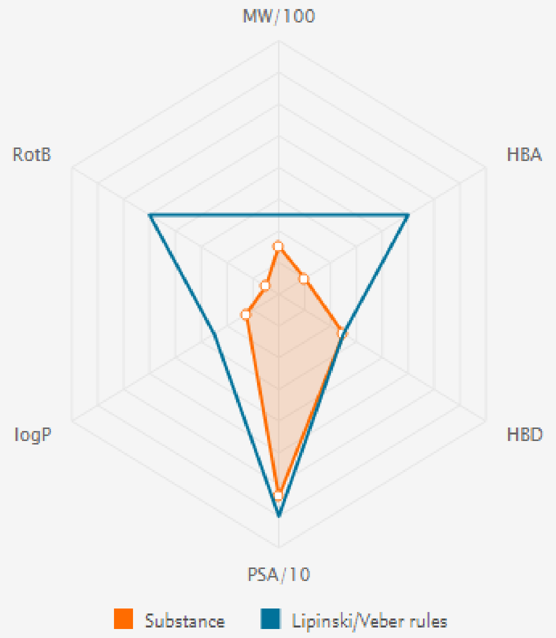

Figure S3. ***In silico*** **drug likeness of** compound **3.**

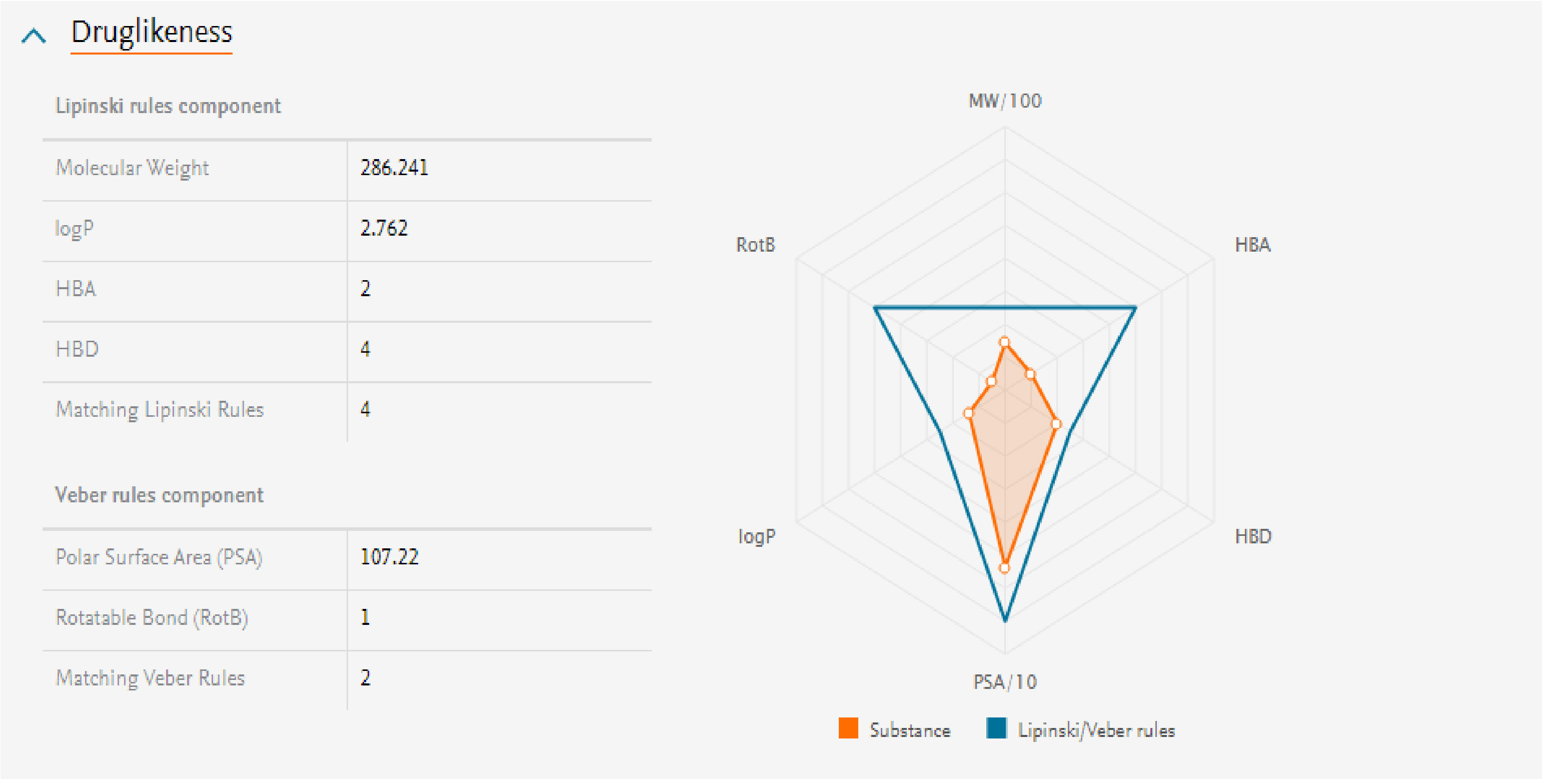

Druglikeness

Lipinski rules component

Molecular Weight	286.241
logP	2.762
HBA	2
HBD	4
Matching Lipinski Rules	4

Veber rules component

Polar Surface Area (PSA)	107.22
Rotatable Bond (RotB)	1
Matching Veber Rules	2

Figure S4. ***In silico*** **drug likeness of** compound **4.**

Druglikeness

Lipinski rules component

Molecular Weight	170.122
logP	1.009
HBA	2
HBD	4
Matching Lipinski Rules	4

Veber rules component

Polar Surface Area (PSA)	97.99
Rotatable Bond (RotB)	1
Matching Veber Rules	2

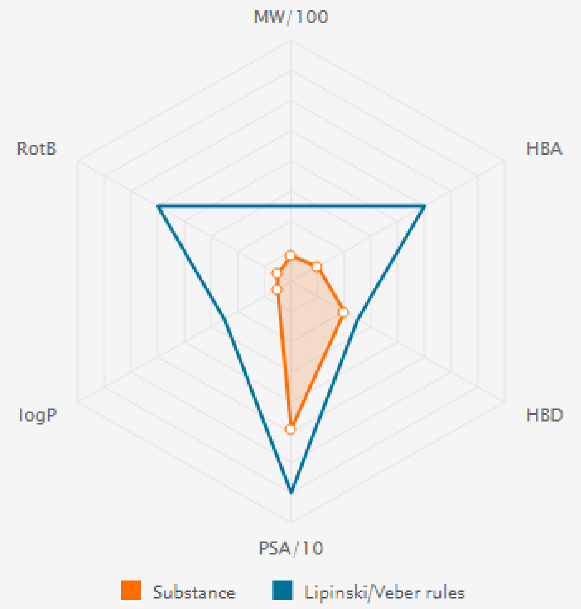

Figure S5. ***In silico*** **drug likeness of** compound **5.**

Druglikeness

Lipinski rules component

Molecular Weight	164.161
logP	1.503
HBA	2
HBD	2
Matching Lipinski Rules	4

Veber rules component

Polar Surface Area (PSA)	57.53
Rotatable Bond (RotB)	2
Matching Veber Rules	2

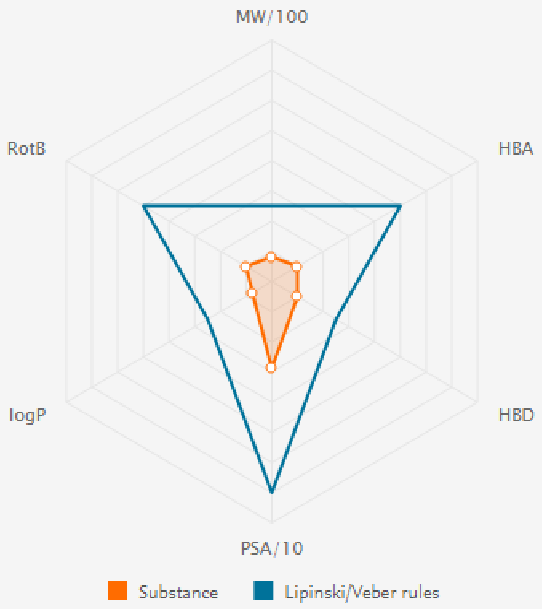

Figure S6. ***In silico*** **drug likeness of** compound **6.**

Druglikeness

Lipinski rules component

Molecular Weight	194.187
logP	1.15
HBA	2
HBD	2
Matching Lipinski Rules	4

Veber rules component

Polar Surface Area (PSA)	66.76
Rotatable Bond (RotB)	3
Matching Veber Rules	2

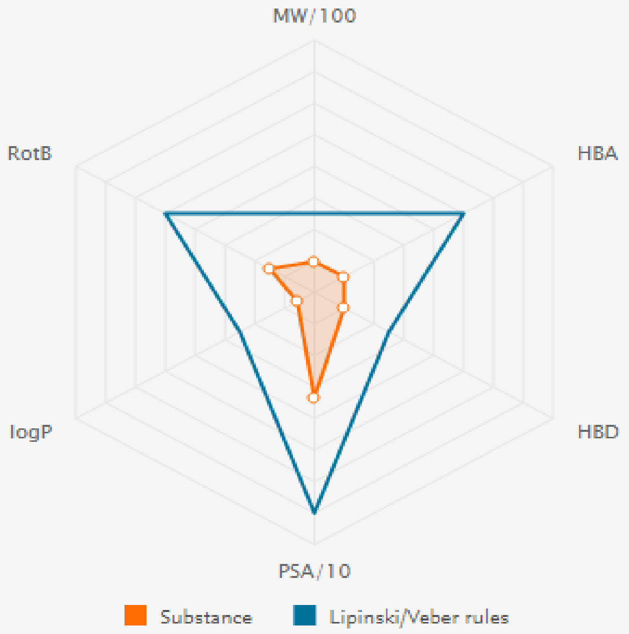

Figure S7. ***In silico*** **drug likeness of** compound **7.**

Druglikeness

Lipinski rules component	
Molecular Weight	180.16
logP	1.258
HBA	2
HBD	3
Matching Lipinski Rules	4

Veber rules component	
Polar Surface Area (PSA)	77.76
Rotatable Bond (RotB)	2
Matching Veber Rules	2

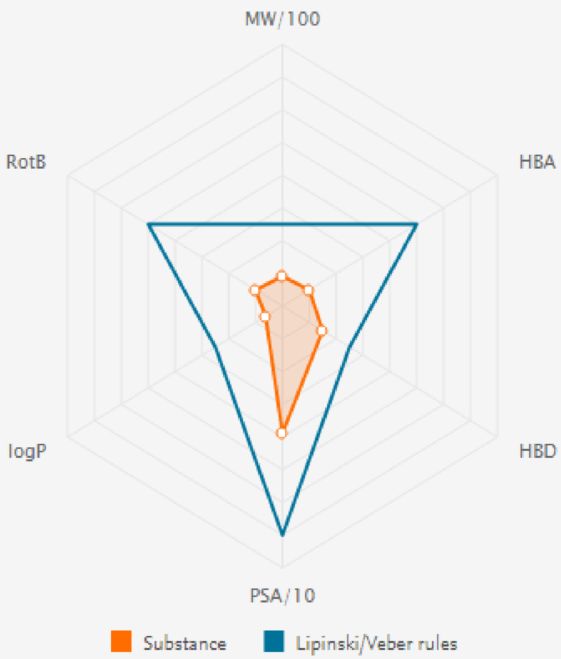

Figure S8. ***In silico*** **drug likeness of** compound **8.**

Druglikeness

Lipinski rules component	
Molecular Weight	154.122
logP	0.986
HBA	2
HBD	3
Matching Lipinski Rules	4

Veber rules component	
Polar Surface Area (PSA)	77.76
Rotatable Bond (RotB)	1
Matching Veber Rules	2

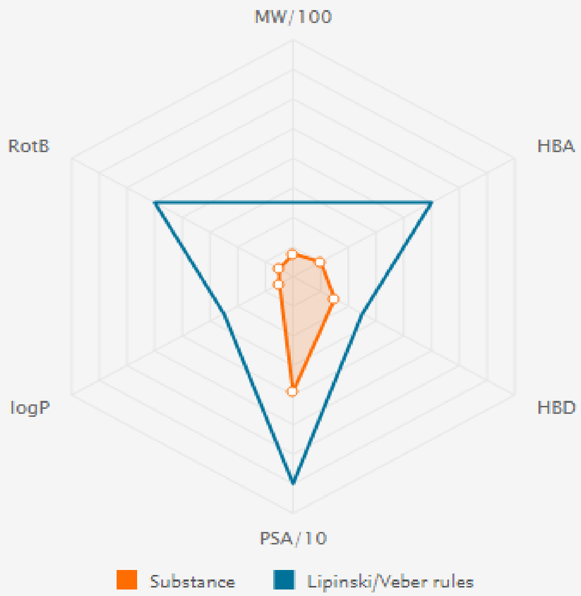

Figure S9. ***In silico*** **drug likeness of** compound **9.**

Druglikeness

Lipinski rules component	
Molecular Weight	148.161
logP	1.909
HBA	2
HBD	1
Matching Lipinski Rules	4

Veber rules component	
Polar Surface Area (PSA)	37.3
Rotatable Bond (RotB)	2
Matching Veber Rules	2

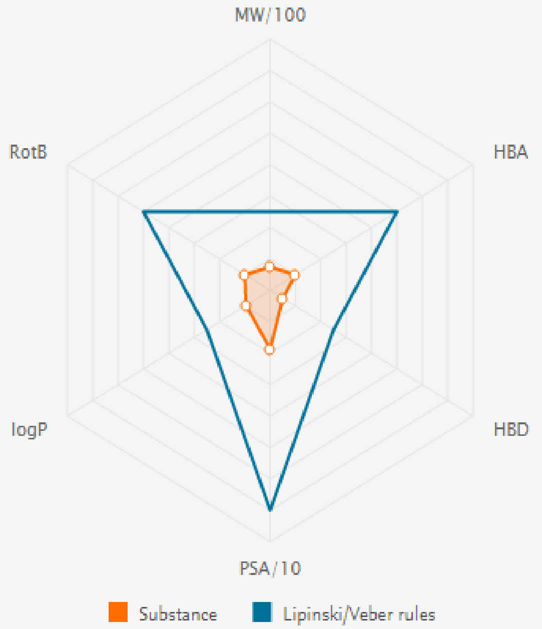

Figure S10. ***In silico*** **drug likeness of** compound **10.**

Druglikeness

Lipinski rules component

Molecular Weight	302.197
logP	1.888
HBA	2
HBD	4
Matching Lipinski Rules	4

Veber rules component

Polar Surface Area (PSA)	133.52
Rotatable Bond (RotB)	0
Matching Veber Rules	2

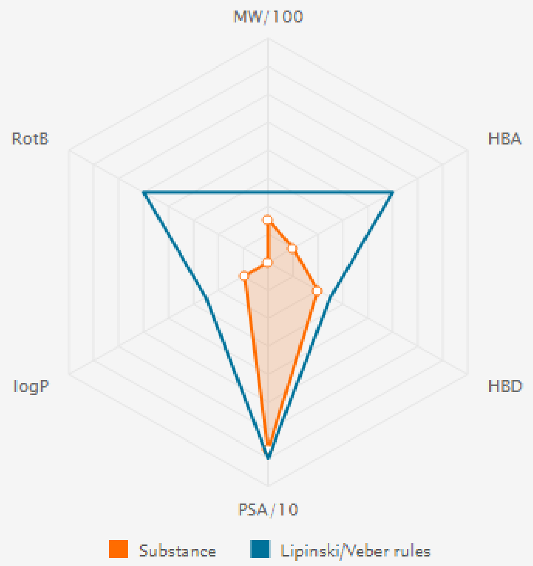

Figure S11. ***In silico*** **drug likeness of** compound **11.**

Druglikeness

Lipinski rules component

Molecular Weight	168.149
logP	0.878
HBA	2
HBD	2
Matching Lipinski Rules	4

Veber rules component

Polar Surface Area (PSA)	66.76
Rotatable Bond (RotB)	2
Matching Veber Rules	2

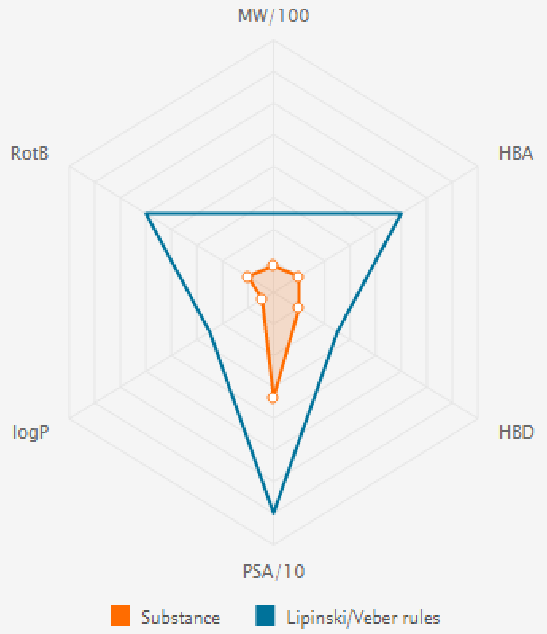

Figure S12. ***In silico*** **drug likeness of** compound **12.**

Druglikeness

Lipinski rules component

Molecular Weight	107.155
logP	1.144
HBA	1
HBD	1
Matching Lipinski Rules	4

Veber rules component

Polar Surface Area (PSA)	26.02
Rotatable Bond (RotB)	1
Matching Veber Rules	2

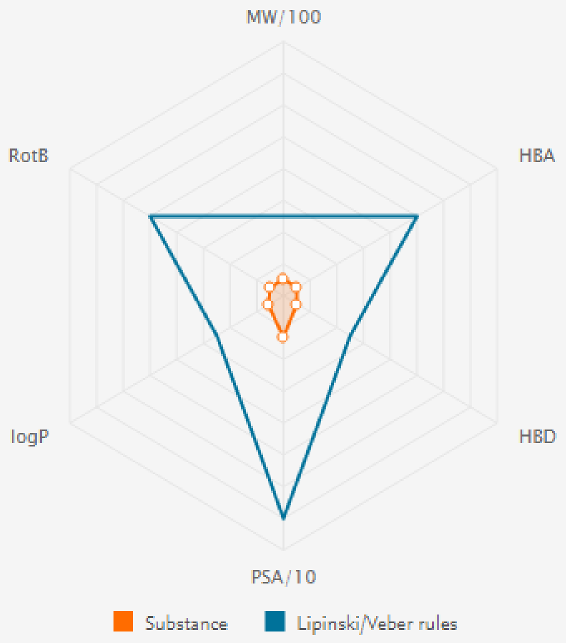

Figure S13. ***In silico*** **drug likeness of** compound **13.**

Druglikeness

Lipinski rules component

Molecular Weight	902.812
logP	-1.947
HBA	20
HBD	14
Matching Lipinski Rules	1

Veber rules component

Polar Surface Area (PSA)	383.36
Rotatable Bond (RotB)	11
Matching Veber Rules	0

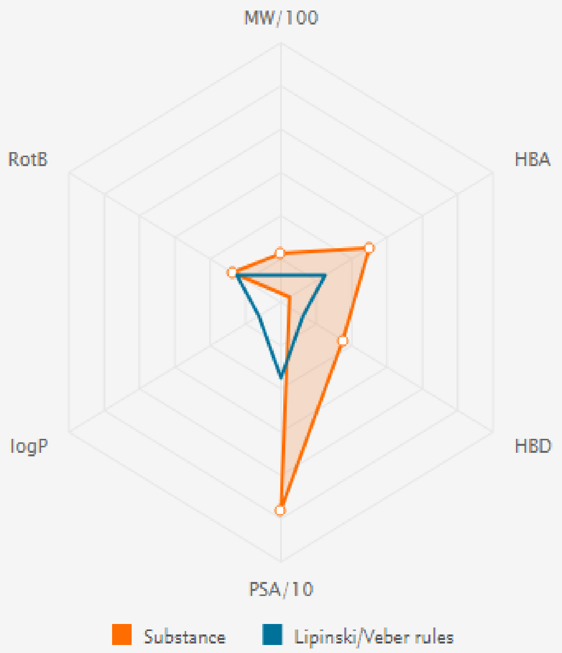

Figure S14. ***In silico*** **drug likeness of** compound **14.**

Druglikeness

Lipinski rules component	
Molecular Weight	311.359
logP	1.807
HBA	5
HBD	3
Matching Lipinski Rules	4

Veber rules component	
Polar Surface Area (PSA)	123.6
Rotatable Bond (RotB)	4
Matching Veber Rules	2

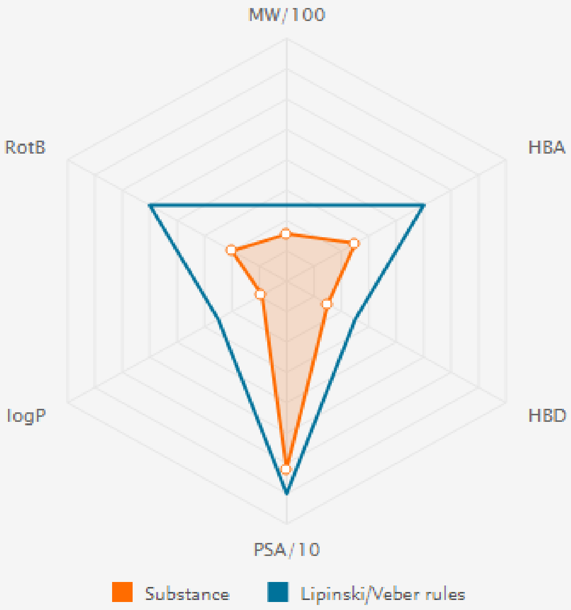

Figure S15. ***In silico*** **drug likeness of** compound **15.**

Druglikeness

Lipinski rules component

Molecular Weight	341.361
logP	1.005
HBA	7
HBD	4
Matching Lipinski Rules	4

Veber rules component

Polar Surface Area (PSA)	117.48
Rotatable Bond (RotB)	7
Matching Veber Rules	2

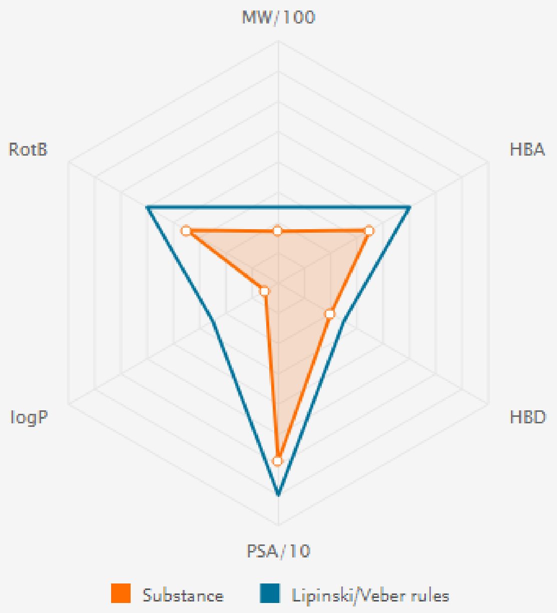

Figure S16. ***In silico*** **drug likeness of** compound **16.**

Druglikeness

Lipinski rules component

Molecular Weight	240.256
logP	1.013
HBA	4
HBD	3
Matching Lipinski Rules	4

Veber rules component

Polar Surface Area (PSA)	79.15
Rotatable Bond (RotB)	2
Matching Veber Rules	2

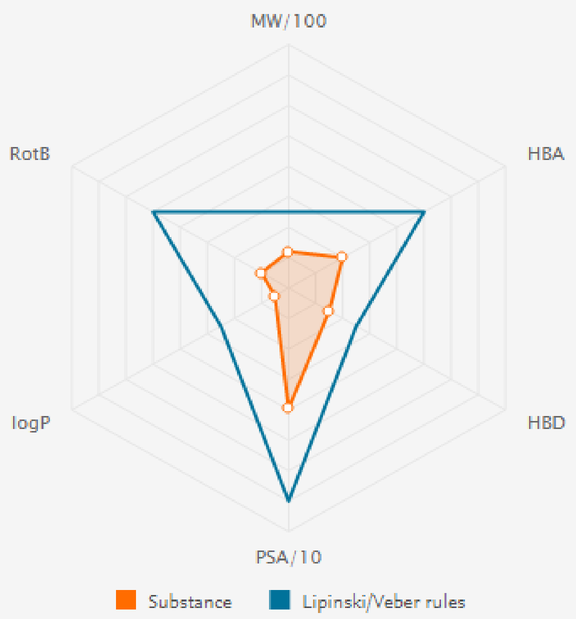

Figure S17. ***In silico* drug likeness of** compound **17.**

Druglikeness

Lipinski rules component

Molecular Weight	297.308
logP	-0.288
HBA	6
HBD	4
Matching Lipinski Rules	4

Veber rules component

Polar Surface Area (PSA)	122.24
Rotatable Bond (RotB)	4
Matching Veber Rules	2

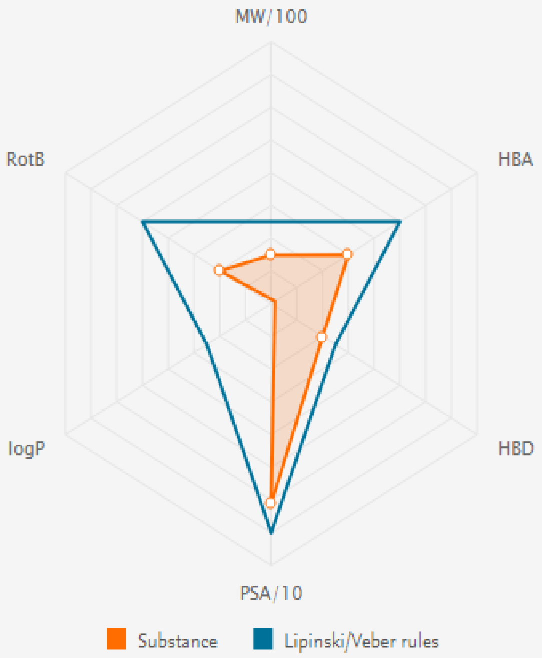

Figure S18. ***In silico*** **drug likeness of** compound **18.**

Druglikeness

Lipinski rules component

Molecular Weight	194.187
logP	1.094
HBA	3
HBD	2
Matching Lipinski Rules	4

Veber rules component

Polar Surface Area (PSA)	66.76
Rotatable Bond (RotB)	0
Matching Veber Rules	2

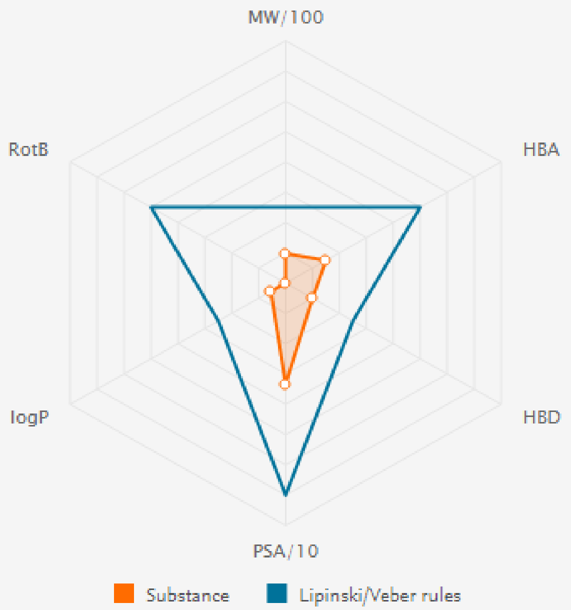

Figure S19. *In silico* drug likeness of compound **19.**

References

1. Gothai, S.; Arulselvan, P.; Tan, W.S.; Fakurazi, S. Wound healing properties of ethyl acetate fraction of Moringa oleifera in normal human dermal fibroblasts. J. Intercult. Ethnopharmacol. 2016, 5, 1–6. https://doi.org/10.5455/jice.20160201055629.
2. Muhammad, A.A.; Pauzi, N.A.S.; Arulselvan, P.; Abas, F.; Fakurazi, S. In Vitro Wound Healing Potential and Identification of Bioactive Compounds from Moringa oleifera Lam. BioMed. Res. Int. 2013, 2013, 974580. https://doi.org/10.1155/2013/974580.
3. Mehwish, H.M.; Liu, G.; Rajoka, M.S.R.; Cai, H.; Zhong, J.; Song, X.; Xia, L.; Wang, M.; Aadil, R.M.; Inam-Ur-Raheem, M.; et al. Therapeutic potential of Moringa oleifera seed polysaccharide embedded silver nanoparticles in wound healing. Int. J. Biol. Macromol. 2021, 184, 144–158. https://doi.org/10.1016/j.ijbiomac.2021.05.202.
4. Govardhan Singh, R.S.; Negi, P.S.; Radha, C. Phenolic composition, antioxidant and antimicrobial activities of free and bound phenolic extracts of *Moringa oleifera* seed flour. *J. Funct. Foods* **2013**, *5*, 1883–1891
5. Singh, B.N.; Singh, B.R.; Singh, R.L.; Prakash, D.; Dhakarey, R.; Upadhyay, G.; Singh, H.B. Oxidative DNA damage protective activity, antioxidant and anti-quorum sensing potentials of Moringa oleifera. *Food Chem. Toxicol.* **2009**, *47*, 1109–1116. https://doi.org/10.1016/j.fct.2009.01.034.
